# Structures of *Plasmodium falciparum* Chloroquine Resistance Transporter (PfCRT) Isoforms and Their Interactions with Chloroquine

**DOI:** 10.1021/acs.biochem.2c00669

**Published:** 2023-02-17

**Authors:** Andreas Willems, Adrian Kalaw, Ayse Ecer, Amitesh Kotwal, Luke D. Roepe, Paul D. Roepe

**Affiliations:** Departments of Chemistry and Biochemistry and Cellular and Molecular Biology, Georgetown University, 37th and O Streets NW, Washington, District of Columbia 20057, United States

## Abstract

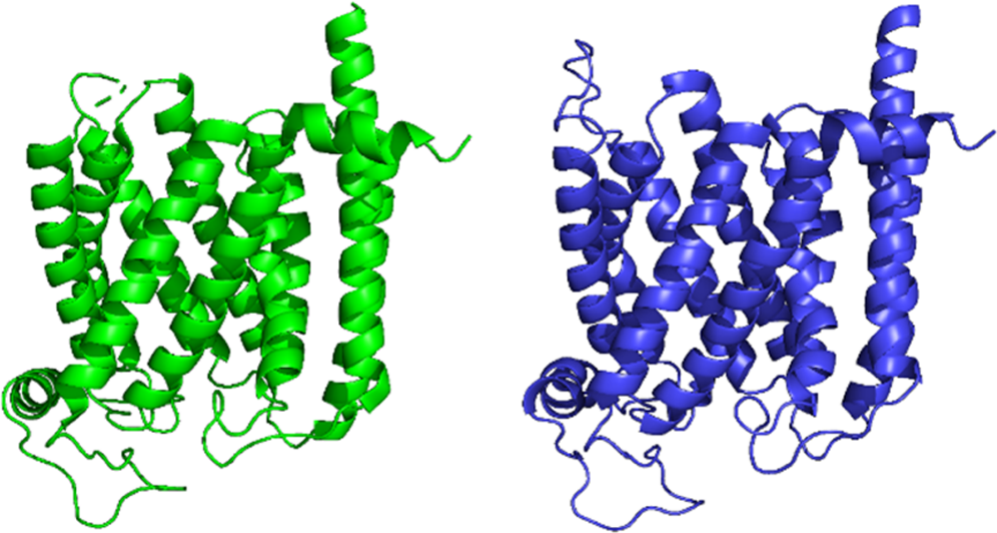

Using a recently elucidated atomic-resolution cryogenic electron microscopy (cryo-EM) structure for the *Plasmodium falciparum* chloroquine resistance transporter (PfCRT) protein 7G8 isoform as template [KimJ.; Nature2019, 576, 315−32031776516 10.1038/s41586-019-1795-xPMC6911266], we use Monte Carlo molecular dynamics (MC/MD) simulations of PfCRT embedded in a 1-palmitoyl-2-oleoyl-*sn*-glycero-3-phosphocholine (POPC) membrane to solve energy-minimized structures for 7G8 PfCRT and two additional PfCRT isoforms that harbor 5 or 7 amino acid substitutions relative to 7G8 PfCRT. Guided by drug binding previously defined using chloroquine (CQ) photoaffinity probe labeling, we also use MC/MD energy minimization to elucidate likely CQ binding geometries for the three membrane-embedded isoforms. We inventory salt bridges and hydrogen bonds in these structures and summarize how the limited changes in primary sequence subtly perturb local PfCRT isoform structure. In addition, we use the “AlphaFold” artificial intelligence AlphaFold2 (AF2) algorithm to solve for domain structure that was not resolved in the previously reported 7G8 PfCRT cryo-EM structure, and perform MC/MD energy minimization for the membrane-embedded AF2 structures of all three PfCRT isoforms. We compare energy-minimized structures generated using cryo-EM vs AF2 templates. The results suggest how amino acid substitutions in drug resistance-associated isoforms of PfCRT influence PfCRT structure and CQ transport.

## Introduction

At least 76 different isoforms of the *Plasmodium falciparum* chloroquine resistance transporter (PfCRT) are expressed in various drug-resistant *P. falciparum* malarial parasites from around the globe. These harbor patterns of multiple amino acid substitutions relative to wild-type PfCRT expressed in drug-sensitive parasites, and they mediate different patterns of drug resistance. How the presumed structural alterations among these isoforms are related to different drug resistance phenomena is poorly understood.

PfCRT was first identified and linked to chloroquine resistance (CQR) via the well-known chloroquine-sensitive (CQS) strain HB3 vs chloroquine-resistant (CQR) strain Dd2 genetic cross.^1^ It was suggested, and later confirmed,^2−4^ that PfCRT-mediated CQR was at least in part due to PfCRT-mediated CQ transport across the malaria parasite digestive vacuolar (DV) membrane. This was consistent with DV localization of drug targets, predicted hydropathy and DV membrane localization of PfCRT, and earlier data that had suggested PfCRT-mediated drug transport.^5,6^ Additional work has shown that CQ transport by PfCRT mediates resistance to the cytostatic (cell growth inhibitory) effects of CQ but is less responsible for resistance to the parasiticidal (parasite kill) effects of CQ^7−9^ thereby linking the former, but perhaps not the latter, to PfCRT-mediated drug transport. Additional amino acid substitutions within CQR-associated PfCRT isoforms mediate resistance to other quinoline-based antimalarial drugs^5,10,11^ as well as reversion back to reduced drug transport linked to CQS-associated isoform transport function.^12^ Further elucidating interaction between PfCRT, CQ, and other drugs remains central to the molecular definition of antimalarial drug resistance phenomena and to the development of improved therapy.

When PfCRT was first identified, only a few isoforms were deduced, including those expressed in laboratory strains HB3 (chloroquine-sensitive; CQS), Dd2 (chloroquine-resistant; CQR), and 7G8 (CQR). In general, the many different PfCRT isoforms that are now known to exist are named for the cognate strains in which they are expressed.^12^ The CQS isolate from which strain HB3 was established was obtained in Honduras, whereas those from which CQR strains Dd2 and 7G8 are derived originate from South East Asia (SEA) and South America (SA), respectively. The PfCRT isoforms expressed in strains Dd2 and 7G8 harbor 8 and 5 amino acid substitutions relative to HB3 PfCRT, respectively. The amino acid sequence of wild-type HB3 PfCRT is the same as that found for 3D7 PfCRT, which is expressed in CQS strain 3D7 derived from an African isolate.

Expression of the Dd2 CQR-associated PfCRT isoform within a CQS parasite without subsequent CQ selection of the engineered clones confers ∼90% of the ∼10-fold shift in CQ IC_50_ that characterizes cytostatic CQR (resistance to CQ growth inhibitory effects) for well-studied laboratory strains of CQR parasites.^13^ Experiments with the same reverse-engineered parasite clones show that expression of Dd2 mutant PfCRT confers <10% of the CQ LD_50_ shifts that quantify resistance to the parasiticidal effects of CQ measured for strains derived from African or S.E. Asian CQR parasites.^7,9^ CQ is growth inhibitory at lower IC_50_ concentrations (nM) but kills parasites at higher parasiticidal LD_50_ doses (μM) that correspond to therapeutic levels measured in patient plasma.^9^ These IC_50_ and LD_50_ are quantified by different assays that measure the relative rate of growth during continuous drug exposure (IC_50_) or outgrowth of parasite populations after bolus dose of higher drug concentrations for a fixed period of time that models peak plasma levels^7^ (see ref (9) for an extended discussion).

In sum, drug resistance mediated by *pfcrt* mutation is complex and likely a reflection of the complex -static and -cidal molecular pharmacology of CQ and other antimalarial drugs that have enjoyed widespread but varied use across the globe for decades.^14^ Presumably, varied use of CQ and other drugs over the past 70–80 years, along with genetically complex drug pharmacokinetics/pharmacodynamics (PK/PD), has selected for a variety of CQR malarial parasites with different levels of CQR and different patterns of drug resistance. These parasites express different mutant PfCRT isoforms^14,15^ that interact with CQ and other drugs in different ways.

Indeed, a quantitative analysis of CQ drug transport mediated by most PfCRT isoforms now known to exist,^12,16^ along with analysis of *pfcrt* transfectants^10^ strongly suggests that substitution of the amino acid residues that characterize different mutant PfCRTs alters binding and transport of CQ, to confer different levels of growth inhibitory CQR. However, detailed structure–function principles for the drug transporter are lacking, and specifically how these amino acid substitutions affect drug binding and transport is unknown. These questions are heightened by some disagreement in the literature over whether CQS-associated PfCRT isoforms mediate drug transport. In brief, drug binding studies using equilibrium binding measurements with [^3^H]-CQ or a CQ photoaffinity analogue clearly showed that CQ binds to wild-type PfCRT under deenergized conditions^2,17^ and studies with yeast expressing PfCRT^12^ or with purified PfCRT reconstituted into proteoliposomes^4^ showed that CQS-associated PfCRT transports drug, albeit with lower efficiency and reduced membrane potential dependence relative to transport mediated by CQR-associated isoforms.^18^ In contrast, transport studies using oocytes injected with *pfcrt* cDNA suggested that wild-type HB3 PfCRT isoform does not transport CQ.^3^ These differences in interpretation may be due at least in part to the fact that yeast and proteoliposome studies used wild-type PfCRT protein whereas oocyte studies used eggs injected with *pfcrt* cDNA that encoded extensively mutated PfCRT.

Recently, an atomic-resolution three-dimensional structure of the 7G8 isoform of PfCRT was solved by Mancia and collaborators.^19^ The structure harbors 10 transmembrane (TM) helical domains and is consistent with earlier studies that defined a CQ binding site for deenergized PfCRT (i.e., PfCRT not exposed to a transmembraneous electrical potential difference or pH gradient as is the case for PfCRT within the live parasite).^17^ This CQ binding site for deenergized PfCRT is near the DV disposed face of PfCRT.^17,19^ The cryogenic electron microscopy (cryo-EM) atomic-resolution structure is not of biologic membrane-incorporated PfCRT, is not energy-minimized, and does not include bound drug, well-resolved N or C termini, or a well-resolved cytosolic loop between TM 2 and 3. Therefore, using this cryo-EM structure as the initial template, and Monte Carlo molecular dynamics (MC/MD) energy minimization calculations for membrane-embedded, solvated PfCRT performed using Desmond,^20^ we deduce structural alterations that occur within the membrane-embedded 10 TM “core” structure of PfCRT upon amino acid substitutions that characterize the evolution of CQS HB3 PfCRT to CQR 7G8 PfCRT and CQS 3D7 PfCRT (identical in sequence to HB3 PfCRT) to CQR Dd2 PfCRT that has occurred in SA and SEA, respectively. Next, using these energy-minimized PfCRT isoform structures, we inventory changes in salt bridges (SB) and hydrogen bonds (HB) and investigate how these might affect PfCRT structure, CQ binding, and CQ transport. We also test these results using MC/MD energy minimization of complete, full-length PfCRT structures determined via AlphaFold artificial intelligence (AFAI) methods recently perfected for proteins.^21^ Importantly, AFAI resolves PfCRT N and C termini and loop 2 structure that were not previously resolved by cryo-EM.^19^ MC/MD energy minimization following AFAI structure determination yields core 10 TM PfCRT structures that are nearly identical to those we find after MC/MD energy minimization of the experimentally determined 7G8 cryo-EM structure. However, the tandem AFAI MC/MD method also reveals additional structure for previously unresolved N and C termini. The results are important for understanding PfCRT structure and function, the mechanism of CQR, and the development of novel second-tier drug therapy active vs CQR malaria.

## Methods

### Protein Mutagenesis and Structure Preparation

An illustration of our computational workflow is shown in Figure S1, and the amino acid differences that distinguish the three PfCRT isoforms studied here are summarized in Table S1. In brief, we first imported the atomic-resolution structure of the 7G8 isoform of PfCRT solved by cryo-EM (PDB code: 6UKJ(19)) into Maestro (Figure S1A).^20^ The amino acid sequences of the other PfCRT isoforms (HB3, Dd2) were generated using Maestro’s Residue and Loop Mutation tool. Each isoform was then prepared using Protein Preparation Wizard^22^ (Figure S1B), which assigned bond orders, removed structure of the F′(ab) fragment used in 7G8 PfCRT cryo-EM structure elucidation,^19^ and added missing hydrogen atoms, loops and side chains using Prime.^23^ Protonation states of ionizable residues were fixed at pH 5.0, near the pH of the DV where PfCRT is found, with ProtAssign.^22^

### Molecular Dynamics

Restrained energy minimization with heavy atoms converged to 0.30 Å was performed using the OPLS4 force field. MC/MD energy minimization for the three PfCRT protein isoforms (HB3, Dd2, 7G8) was performed either with (Figure S1F) or without (C) docked drug. In both cases, the Maestro System Builder Panel was first used to embed each isoform within a 1-palmitoyl-2-oleoyl-*sn*-glycero-3-phosphocholine (POPC) membrane. The boundary conditions were set as an orthorhombic box expanding 10 Å beyond the protein in the *X*, *Y*, and *Z* dimensions (where the membrane defines the *X*, *Y* plane), solvated with simple point-charge (SPC) water, and system-neutralized with chloride ions. MC/MD was then performed using Desmond^23^ run within “Maestro”.^24^ Each calculation began with a default system relaxation protocol followed by simulation in an isothermal, isobaric NPT ensemble with constant particle number (*N*), pressure (*P*; 1.01325 bar), and temperature (*T*; 310 K). Simulation event analysis and interaction diagrams were then used to analyze resultant protein structure and protein–drug interactions (Figure S1D,G, respectively). Undocked and docked MC/MD simulations were 10 and 100 ns, respectively.

To display and analyze PfCRT isoforms, as well as their CQ bound states, hierarchical clustering was performed with the Desmond Trajectory Clustering tool.^25^ Clusters were generated for each MC/MD trial and for all combined trials by sampling each frame using all atoms as the root-mean-square deviation (RMSD) matrix. Water and POPC membrane were manually removed with Maestro for ease of visual comparison (see the Results section).

Three independent simulations, each with randomized starting velocity, were used to generate three energy-minimized structures for each membrane embedded isoform (7G8, HB3, Dd2) either +(100 ns) or −(10 ns) bound drug (Figure S1C,F). Convergence was typically observed within 2–4 ns (Figure S2). All frames from all three simulations of a given type were then clustered (averaged) together. We call these “EMMD” structures since the cryo-EM 7G8 PfCRT structure is the initial template. To compare PfCRT isoforms, all-atom RMSD were computed using Maestro superposition, and to further visualize local structural differences, the PyMOL script ColorByRMSD^26^ was applied. This superimposes two structures by minimizing paired α carbons and colors them to indicate minimum and maximum pairwise RMSD, respectively (see the Results section).

### Drug Docking

The drug structure was imported into Maestro from PubChem or constructed using 2D Sketcher.^24^ Drugs were then prepared for energy minimization calculations using LigPrep. In this step, Epik^27^ generated possible tautomeric and protonation states, and the drug ligands were then energy-minimized using the OPLS4 force field.

Sitemap^28^ was initially used to identify likely drug binding sites for PfCRT EMMD structures (Figure S1E). We filtered to five sites requiring at least 20 site points of contact. As described in the Results section, drug docking used a more restrictive definition of hydrophobicity, a standard grid, and cropped site maps at 8 Å from the nearest site point; “shallow” sites were not considered.^29^

Grids were then constructed and calculated for later use in drug docking. To perform drug docking guided by previous drug photoaffinity labeling studies,^17^ a grid box that enclosed the known drug binding site was defined and a glide grid was constructed.^29,30^ For all PfCRT isoforms, the largest possible drug binding sites reported by SiteMap were at the DV opening of the protein, overlapping or immediately adjacent to the mass spectrometry-mapped position of the previously identified deenergized CQ binding site.^17^

Additional drug docking was then performed with Glide at extra precision with flexible ligand sampling; post-docking minimization was performed with a 0.50 kcal/mol threshold for rejecting a minimized pose.^30^ Initial poses were then used for induced fit docking, which refined the original pose with Prime to account for protein structural flexibility in the presence of docked drug. Residues within 5 Å of ligand poses were refined, and structures within 30 kcal/mol of the highest-ranked protein structure (based on the ligand binding affinity and internal strain energy) were returned to Glide for docking with extra precision. From the resulting structures that included docked drug ligand, the highest rank drug pose with the strongest protein–ligand interaction (highest binding affinity and lowest energetic cost due to torsional strain) was merged with the protein structure to assemble the initial protein–drug complex. This complex was then embedded in membrane, solvated, and refined using MC/MD energy minimization once again as described above (Figure S1F).

### AlphaFold

AlphaFold artificial intelligence (AFAI) has revolutionized the analysis of protein structure–function relationships.^21,31,32^ In brief, 3D atomic-resolution structures are generated within hours from primary amino acid sequence data, using all available pdb files of all known atomic-resolution protein structures as the artificial intelligence (AI) training set. Consistent with what we also now find for PfCRT, detailed comparison between AFAI structures and structures solved experimentally by either single-crystal X-ray or cryo-EM diffraction methods shows that, remarkably, for proteins without co-factors or quaternary structure, AFAI structures are essentially identical to those determined experimentally (e.g., see RMSD-based comparisons for PfCRT, below). First, we compared AFAI PfCRT structures generated using a pdb training data set that either did or did not include the cryo-EM experimentally derived PfCRT structure^19^ and found no significant difference between the two (see below), further emphasizing the distinction between AI training vs homology modeling methods.^21^ To derive AFAI structures for PfCRTs (Figure S1H) AlphaFold v2.1.2^21,32^ was installed from the git repository (https://github.com/deepmind/alphafold) onto a customized computer (see below) using the script provided in the repository. The entire pdb file AlphaFold2 (AF2) database was downloaded to a 2.5 terabyte (TB) dedicated partition on a 4 TB solid-state drive (SSD), while the AlphaFold2 (AF2) python scripts and associated files were saved to a 1.2 TB dedicated working partition on the same SSD. To generate PfCRT isoform structures (full length 7G8, HB3, and Dd2 PfCRTs), the protein fasta files were first downloaded from PlasmoDB (7G8: Pf7G8_070014400; HB3: PfHB3_070013000; Dd2: PfDd2_070013200). The parameter max_template_date was set to 2022-02-14 unless otherwise noted. From the AF2 output folder three different levels of structures are generated: unrelaxed, relaxed following the Amber relaxation procedure, and ranked structures which are the relaxed structures ranked by model confidence. From the generated structures, “ranked_0.pdb” (the highest-ranked structure based on model confidence) was then optimized and energy-minimized following the procedures described in the Molecular Dynamics section to yield energy-minimized structures using the Alphafold structure as the starting point, we call these “AFMD” structures (Figure S1J) to distinguish them from EMMD structures (Figure S1C).

As mentioned, due to the presence of the experimentally derived cryo-EM 7G8 PfCRT structure in the PDB database (6UKJ^19^), which would therefore lie within the default AI training set, we also solved for AFAI PfCRT isoform structures using a truncated database training set that excluded the 7G8 PfCRT cryo-EM structure. This was done by setting the max_template_date parameter to 2019-10-01, 5 days prior to the 7G8 PfCRT cryo-EM structure being deposited. There were no differences between structures solved using AF2 software “trained” with either training set (see the Results section).

### Structural Analysis

Thus, MC/MD energy-minimized structures for HB3, 7G8, and Dd2 PfCRT isoforms embedded in membrane were solved using either a cryo-EM PfCRT structure (“7G8^EM^”)^19^ or AF2-generated structures (“HB3^AF^”, “7G8^AF^”, or “Dd2^AF^”) as the initial templates. These are referred to as EMMD and AFMD structures, respectively (Figure S1C,J). The energy-minimized structures were subsequently imported into PyMOL v2.4.0 (Schrödinger, LLC, New York, NY) and VMD^33^ software for hydrogen bond (HB) and salt bridge (SB) network inventory. For each charged residue (D,E,R,K,H), the nearest, oppositely charged amino acid residue was found, and the distance was measured from nitrogen on the positive residue to the closest oxygen on the negative residue; the minimum criterium for an SB was defined as at least one D or E side-chain carbonyl oxygen being within 4 Å from an R,K, or H side-chain nitrogen.^34^ HB networks were initially identified using the PyMOL script list_hb.py;^35^ the heteroatom-to-heteroatom distance cutoff was initially set to 3.2 Å (see the Results section) and the maximum angle was set to 180°.^36^ SBs and HBs in the simulation trajectory files were also found using Visual Molecular Dynamics’s (VMD)^33^ SB and HB analysis tools. In short, the clustered simulation files were imported into VMD by opening the out.cms file and then loading the associated Desmond trajectory file. The HBs were found by sampling the entire protein and looking for HBs where the heteroatom distances were within 3.2 Å and the bond angle 180 ± 45°. Detailed bond data were calculated for residue pairs, giving a list of residue pairs where side-chain or peptide backbone atoms interact as well as the percentage of time the residue pair is within a given distance and angle (see the Results section). SBs were found using the SB tool^33^ and heatmaps were generated using R studio with the tidyr package.^37^ Each protein isoform was examined in its entirety using an SB heteroatom interaction cutoff of 4 Å. The SB data were written out as a single frame vs distance file for each residue pair. These files were then imported into Excel where the amount of time the pairs were within the 4 Å cutoff was calculated and expressed as a percentage of all frames. Alternatively, heatmaps summarizing pair interaction were generated using VMD.^33^

Results from scripts were verified by direct visual examination using PyMol. Key SB or HB interactions that are affected by PfCRT mutation or drug binding were analyzed across simulation time.

### Computer Design and Hardware

To perform cost-effective MC/MD calculations as above during a laboratory access restricted Covid-19 pandemic, we built a custom server for running either remote or local computational modeling tasks. The assembled server uses supported Ubuntu (20.04.1 LTS) and is equipped with an Intel Xeon W-2145 8-core, 16-thread CPU running at 3.7 GHz on an Asus workstation motherboard (Asus WS C422 PRO/SE) and 32 GB of DDR4 ECC memory. MC/MD calculations are performed on a dedicated PNY Nvidia Quadro RTX 5000 GPU with 16 GB GDDR6 memory and a total of 3504 GPU cores. A smaller HP Nvidia Quadro P620 GPU was dedicated to display output. The system runs on a 240 GB SATA SSD that allows fast iterative storage of current job results, while final structure data are stored on a 4 TB local HDD for detailed analysis. All data are archived using an external 8 TB HDD as well as a cloud account hosted on Google Drive, to which the authors are happy to provide access upon request (see the Supporting Information (SI)).

To increase performance and allow for solution of structures using AlphaFold2 (AF2) software, an additional 32 GB of DDR4 ECC memory was added to the system along with a 4 TB SSD, which was dedicated to all tasks related to AF2. The 4 TB SSD was partitioned into a database partition of 2.5 TB and a working partition of 1.2 TB.

## Results

### 3D Structural Alignments

**Figure 1:**
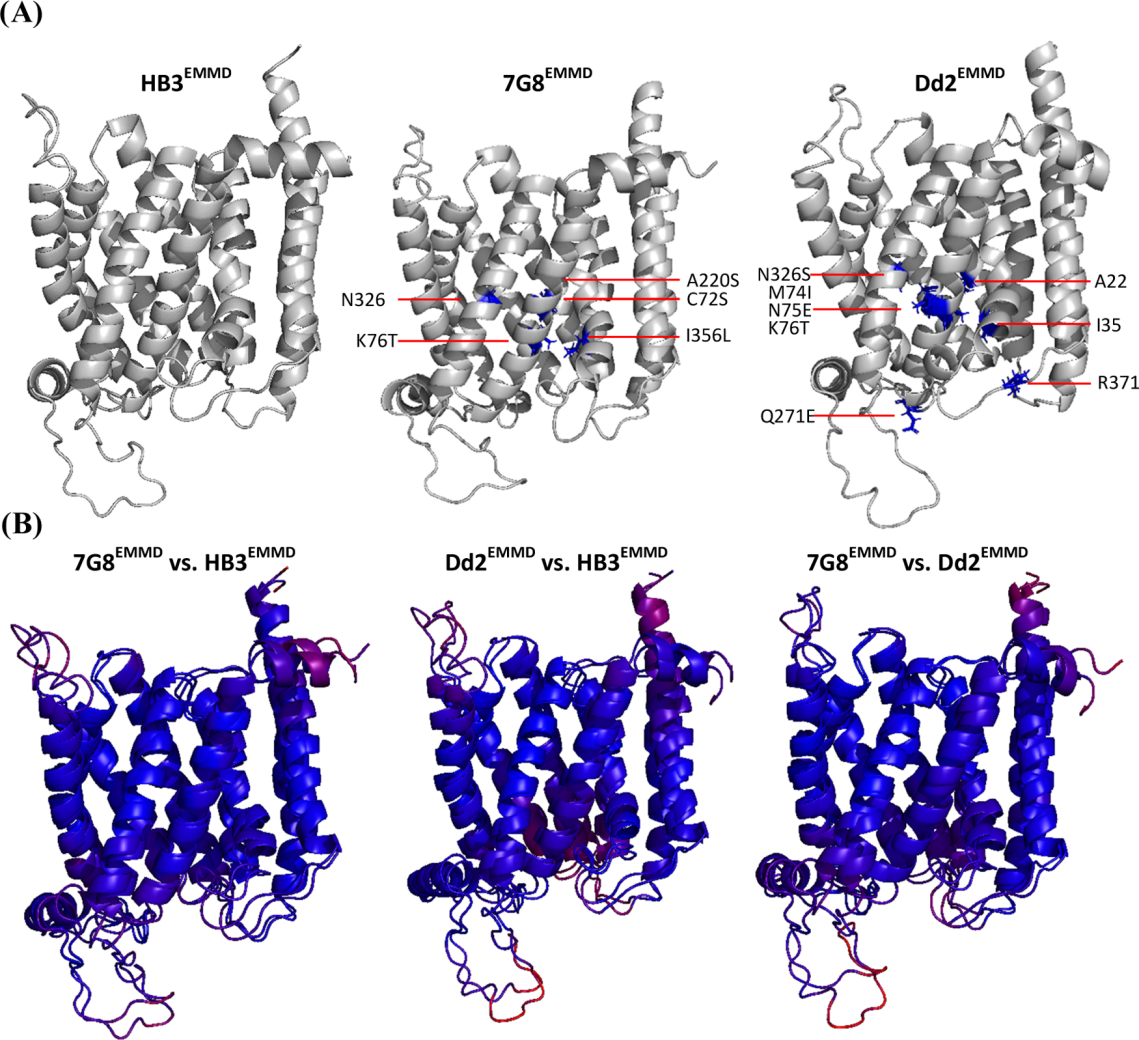
(A, top) MC/MD energy-minimized structures of membrane-embedded HB3 (left), 7G8 (middle), and Dd2 (right) PfCRT isoforms solved using 7G8^EM^ PfCRT as the initial template. Amino acid residues in blue (7G8^EMMD^, middle, Dd2^EMMD^, right) are those that differ relative to HB3 and are labeled. For transmembrane TM helical strands numbering scheme, refer to Figure S7. (B, bottom) RMSD comparisons between 7G8^EMMD^ and HB3^EMMD^ (left), Dd2^EMMD^ and HB3^EMMD^ (middle), and 7G8^EMMD^ and Dd2^EMMD^ (right). Local structural differences are highlighted using ColorByRMSD, blue and red indicate the minimum (0.24, 0.15, and 0.11 Å from left to right) and maximum (9.55, 11.85, and 12.50 Å from left to right) pairwise RMSD, respectively; all-atom average RMSDs are 1.70, 2.36, and 2.17 Å left to right. Each structure is the average of all clustered frames for three independent 10 ns Desmond simulations. All isoforms are shown in the same orientation with the cytosol above the protein and the digestive vacuole lumen below the protein.

Clustered MC/MD energy-minimized structures for each membrane-embedded PfCRT isoform studied here (“HB3” PfCRT [expressed in CQS parasites], “Dd2” [expressed in CQR parasites from SEA], and “7G8” [expressed in CQR parasites from SA]), were first generated using the published cryo-EM structure of 7G8 PfCRT (7G8^EM^) as template (see the Methods section) and are shown in Figure 1.

Overall the three EMMD structures for the three PfCRT isoforms are very similar (Figure 1B). To validate our approach and to identify minor differences among the isoforms, the structures (e.g., averaged across three independent MC/MD simulations) as well as each and every energy-minimized EMMD replicate for a given isoform were separately aligned vs each other (Figure 1B and Table 1).

**Table 1:** (A) Summary of RMSD Comparison between 7G8^EM^ (cf. Ref (19)) and 7G8^EMMD^ Structures, between Other EMMD vs 7G8^EMMD^ Structures, and between Each Isoform AFMD vs EMMD Structure for the Different Isoforms;^a^ (B) Representative RMSD Comparison between Different MC/MD Energy Minimization Simulation Trials for the Same Isoform^b^

(A)				
	with loops		without loops	
	all-atom	α carbon	all-atom	α carbon
7G8^EM^ vs 7G8^EMMD^	1.78	1.47	1.69	1.40
7G8^EMMD^ vs HB3^EMMD^	2.38	1.70	2.15	1.46
7G8^EMMD^ vs Dd2^EMMD^	2.71	2.17	2.31	1.79
Dd2^EMMD^ vs HB3^EMMD^	2.96	2.63	2.61	2.03
HB3^EMMD^ vs HB3^AFMD^	2.86	2.21	2.28	1.64
7G8^EMMD^ vs 7G8^AFMD^	3.74	3.01	3.21	2.58
Dd2^EMMD^ vs Dd2^AFMD^	3.01	2.38	2.55	1.88

(B)				
	with loops		without loops	
	all-atom	α carbon	all-atom	α carbon
HB3^1^ vs HB3^2^	2.63	2.04	2.04	1.37
HB3^2^ vs HB3^3^	2.32	1.66	1.84	1.21
HB3^1^ vs HB3^3^	2.41	1.92	1.92	1.33

a An EM superscript indicates the cryo-EM 7G8 PfCRT structure,^19^ and the EMMD superscript indicates structures derived using the cryo-EM structure as initial template (see text). The AFMD superscript indicates that the structure is derived from AF2 artificial intelligence software followed by MC/MD energy minimization of the membrane-embedded AF2 structure (cf. Figure 3). All MC/MD energy-minimized structures are the result of three independent 10 ns simulations, which were clustered using all frames. All values are given in angstroms. All-atom and α carbon indicates which atoms were used in the RMSD calculation.

b Similar values are seen for the other possible comparisons for the other isoforms, and statistically significant higher RMSD are seen for comparisons between individual trials for different isoforms (not shown, see text). The superscript indicates the trial number. All measurements were confirmed manually using PyMOL (see text).

Regions of variation were initially visualized using ColorByRMSD (e.g., Figure 1B). We tested whether energy-minimized replicates for a given isoform (e.g., MC/MD trial 1 for HB3^EMMD^ vs trial 2 for HB3^EMMD^; Table 1B and Figure S3) were more similar as measured by all-atom RMSD than comparisons between trials for different isoforms (e.g., any trial of HB3^EMMD^ vs any trial of Dd2^EMMD^). For three 10 ns simulations with each of three isoforms, the average all-atom RMSDs were significantly lower between different replicates for a given isoform than for any comparison between any trial for different isoforms (*p* < 0.05 for a two-tailed *t* test; Table 1 and Figure S3). Any two different replicates for the same isoform (e.g., HB3) had all-atom RMSD of ≤2.63, (≤2.04 with helix-connecting loops removed), and even lower α-C RMSD (Table 1B), while comparison between replicates for different isoforms had all-atom RMSD of ≤5.40, (≤5.11 with helix-connecting loops removed [data not shown]).

Pairwise comparison also tested whether RMSD of the clustered structures (average across all frames collected from three independent MC/MD simulations) for different isoforms were related to the number of amino acid differences between them. There are 5, 7, and 8 differences between 7G8 and HB3, Dd2 and 7G8, and HB3 and Dd2 isoforms, respectively (Table S1). Correspondingly, RMSD for the three comparisons is 2.38, 2.71, and 2.96 Å, respectively (Table 1A). Taken together, these data strongly validate our approach of in silico mutagenesis of 7G8^EM^ followed by MC/MD energy minimization as outlined in the Methods section to generate HB3^EMMD^, 7G8^EMMD^, and Dd2^EMMD^ structures (Figure 1).

**Figure 2:**
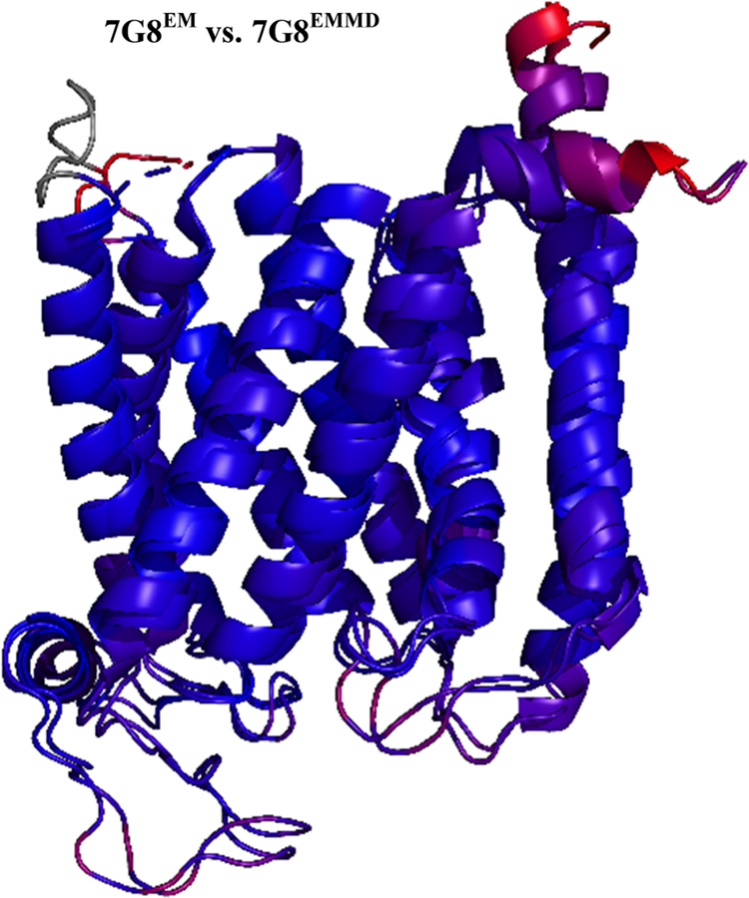
RMSD comparison between static cryo-EM (7G8^EM^)^19^ and membrane-embedded energy-minimized 7G8^EMMD^ structures. Regional differences were compared using ColorByRMSD; blue and red indicate the minimum (0.18 Å) and maximum (7.48 Å) pairwise all-atom RMSD, respectively; the average RMSD is 1.47 Å.

Upon further inspection, we find that clustered EMMD structures for HB3 (Figure 1A, left) 7G8 (Figure 1A, middle), and Dd2 (Figure 1A, right) PfCRT isoforms show small differences in local structure. These are analyzed below. As described in the Methods section, these energy-minimized structures were solved for PfCRT embedded within a POPC membrane using the experimentally determined cryo-EM structure of 7G8 PfCRT (7G8^EM^) as the initial template. To first test the effect of energy minimization calculations alone, Figure 2 shows RMSD comparison between 7G8^EM^ PfCRT^19^ and the cryo-EM structure after membrane embedding and MC/MD energy minimization [“7G8^EMMD^”].

As expected we find that the structures are similar with minor differences found at the DV disposed loop connecting TM7 to JM2 (bottom left, Figure 2), and the cytosolic ends of the N and C termini (top right, Figure 2). These small structural changes found for loop and termini regions (red, Figure 2) are likely due to intrinsic flexibility within these domains that is also evident from frame-by-frame inspection of any one MC/MD simulation (not shown).

Regardless, as expected, upon mutation in silico followed by MC/MD energy minimization, the core 10 TM structures obtained for each of the three membrane-embedded isoforms using 7G8^EM^ as template are quite similar (Figure 1B) but with local structural differences as discussed below.

**Figure 3:**
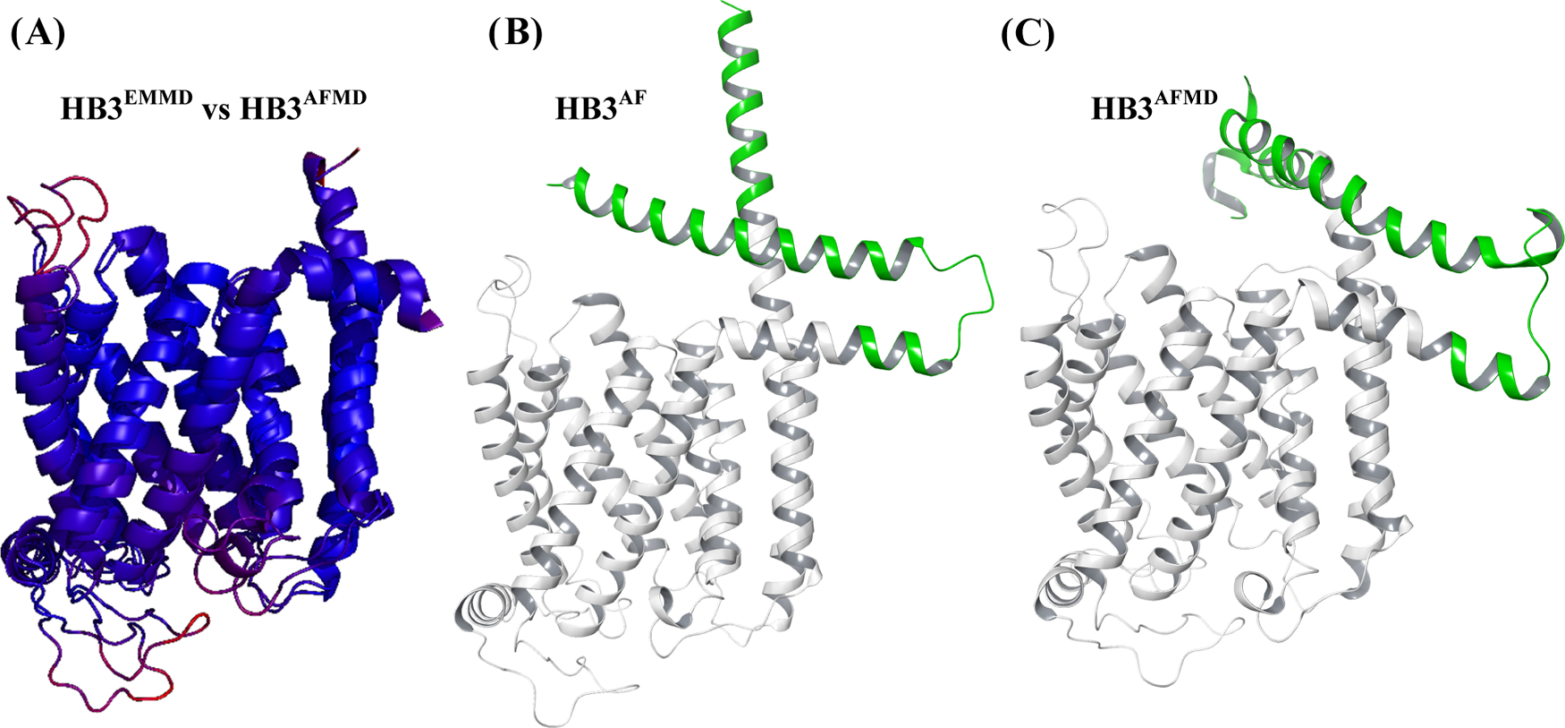
(A) RMSD comparison between 10 TM core HB3^EMMD^ and HB3^AFMD^ structures. 10 TM core regional differences between the two were compared using ColorByRMSD; blue and red indicate minimum (0.13 Å) and maximum (11.71 Å) pairwise all-atom RMSD, respectively; the average RMSD is 2.21 Å. (B, C) Visualization of resolved regions of 7G8^EM^ (shown in white, which is nearly identical to blue in (A)) vs additional PfCRT domains solved by AF2 for (B) HB3^AF^ and upon application of tandem AF2-MC/MD methods for (C) HB3^AFMD^ (shown in green). The HB3^EMMD^ and HB3^AFMD^ structures ((A) and (C), respectively) are both the average clustered structure of three separate 10 ns simulations.

To further test the precision and accuracy of the energy minimization procedure, Figure 3 shows RMSD analysis for HB3 PfCRT structures solved after in silico substitution of 7G8^EM^ PfCRT followed by membrane embedding and MC/MD (to generate “HB3^EMMD^”) vs HB3 PfCRT solved by established AlphaFold2 AI methods (HB3^AF^)^21^ before (Figure 3, middle) or after (Figure 3, right) membrane embedding and MC/MD energy minimization (to yield “HB3^AFMD^”).

We find that the HB3^EMMD^ and HB3^AFMD^ 10 TM core PfCRT structures are similar (Figure 3A, left, blue) again with some small variation in flexible loop regions. Similar results are found for the 10 TM core Dd2 and 7G8 EMMD vs AFMD structures (Figure S4). However, as shown (Figure 3B, green), and as shown previously for other CRT orthologue structures solved by AFAI,^38^ the AF2 algorithm reveals additional loop and N and C termini structure that is not resolved within 7G8^EM^ solved with cryo-EM methods.^19^ For example, much of the N and C termini as well as cytosolically disposed “loop 2” (connecting TM helices 2 and 3) were not resolved for 7G8^EM^ presumably due either to masking by bound F′(ab) used in solving the cryo-EM structure and/or the intrinsic flexibility of these regions;^19^ nonetheless, these regions are resolved in the AF2 structures computed here and elsewhere^38^ (Figure 3B, green). We note that routine display of 7G8^EM^ in programs such as PyMol or Maestro includes a truncated primary amino acid sequence (e.g., missing residues 1–46 [N terminus], 406–424 [C terminus], and 112–124 [most of cytosolically disposed loop 2]) whereas solving PfCRT structure using AF2 incorporates the complete primary amino acid sequence. Hence, termini and loop 2 structure are not resolved for HB3^EMMD^ or Dd2^EMMD^ either (Figure 1), as these were generated using 7G8^EM^ as the initial template (see the Methods section), but are resolved for HB3^AFMD^ and Dd2^AFMD^, which are generated from HB3^AF^ (Figure 3B) and Dd2^AF^, respectively (Figure S1). Interesting additional features absent from 7G8^EM^ and the three EMMD structures (not shown, cf. Figure 3B caption) but revealed upon MC/MD energy minimization of the three AF2-generated structures are described at the end of the Results section. Regardless, analysis of the respective core 10 TM domains shows they are very similar for all AFMD or EMMD PfCRT isoform structures studied here (Figures 3 and S4 and Table 1).

### Analysis of Predicted Salt Bridges (SB) and H Bonds (HB)

Table S1 shows the amino acid differences that distinguish HB3 vs 7G8 vs Dd2 PfCRT isoforms. Both the Dd2 and 7G8 isoforms lack K76 found in HB3 PfCRT and also harbor several other amino acid substitutions. The K76T substitution is particularly common for CQR conferring isoforms; however, we note that some PfCRTs may harbor K76T after also acquiring another “second-site revertant” amino acid substitution that then abolishes increased CQ transport function more typically found for K76T-containing, CQR-associated isoforms, converting the protein back to a PfCRT that transports at or below levels found for HB3 PfCRT.^12^ This predicts that parasites harboring such second-site revertant substitutions along with K76T may not have CQR phenotypes.^39^

Regardless, Table 2 lists key salt bridges (SB) consistently found for the three isoform EMMD structures across ≥50% of simulation time for at least one isoform, grouped by the isoform(s) in which they are present, and lists where the involved residues are located (we use the common “L1, L2” nomenclature, etc. to denote loops 1, 2, etc. that connect TM 1 and 2 and TM 2 and 3, respectively^19^).

**Table 2:** Summary of Key Salt Bridges (SB) Found for 7G8^EMMD^, Dd2^EMMD^, and HB3^EMMD^ PfCRTs Present for ≥50% of MC/MD Simulation Time for at Least One Isoform^a^

	salt bridge	residue 1	residue 2
7G8, HB3, and Dd2	K53/D57	JM1	JM1
	K85/D311	L1	L7
	R231/D137	TM6	TM3
	K200/E204	*TM5*	L5
	K236/E232	*TM6*	TM6
HB3 and Dd2 only	R374/D377	L9	*TM10*
HB3 only	*K76*/D329	TM1	TM8
7G8 only	R392/E54	JM10	TM1
Dd2 only	R392/E399	TM10	*TM10*

a The column entitled “salt bridge” indicates the participating residues, and the columns entitled “residue 1” and “residue 2” indicate the location of the participating residues. SB is defined as ≤4 Å between the heteroatoms of the side chains. Data are the average of three independent 10 ns simulation for each isoform. A table showing lifetimes of all SB found in ≥10% of simulation time for at least one isoform is found in the Supporting Information (Table S2). Residues that are mutated in different isoforms are *italicized*.

A particularly interesting isoform-specific SB is formed between K_76_ and D_329_, which is only seen for HB3 PfCRT, since as mentioned, 7G8 and Dd2 PfCRTs harbor K76T substitution, as do other known CQR-associated isoforms (cf. ref (12)). Additional isoform-specific SB that are either directly or indirectly due to amino acid substitutions across the isoforms are described below, and Table S2 provides a complete listing of all deduced SB that exist ≥10% of all simulation time in at least one of the isoforms. The distance limit for an energetically favorable SB was defined as ≤4 Å from N on the positive residue to O on the negative.^34^

We note that for some key SB (e.g., that involving K_76_ and D_329_), a computed average distance for the SB across all simulation frames would be somewhat misleading since frame-by-frame inspection shows the bridge is either present (e.g., the distance is clearly <4.0 Å) or is not, not that SB distance varies smoothly over a range (see Figure S5).

Tables 3 and S3 are analogous to Tables 2 and S2 except that they summarize computed side chain–side chain hydrogen bonds (HB) that are common vs unique for CQS vs CQR isoforms.

**Table 3:** Summary of Key Side Chain–Side Chain HB Found for 7G8^EMMD^, Dd2^EMMD^, and HB3^EMMD^ PfCRTs.^a^

isoform	hydrogen bond	residue 1	residue 2
7G8, HB3, and Dd2	Y68/D329	TM1	TM8
	Y264/S90	**L7**	*TM2*
	S227/D137	TM6	TM3
	R231/D137	TM6	TM3
	N214/T151	*TM6*	**L3**
	N154/E198	TM4	TM5
	N183/N395	*TM5*	*TM10*
	K200/E204	*TM5*	**L5**
	N214/N209	*TM6*	**L5**
	R231/N228	TM6	TM6
	K236/E232	TM6	*TM6*
	N246/S334	TM7	TM8
	Y361/D377	*TM9*	*TM10*
HB3 and 7G8	*N75*/D329	TM1	TM8
	Y89/D310	**L1**	**L7**
	R150/E208	**L3**	**L5**
	T152/E207	TM4	**L5**
	S341/T344	**L8**	*TM9*
7G8 and Dd2	Y62/E54	TM1	JM1
	H97/*D326(7G8)* or H97/*S326(Dd2)*	TM2	TM8
HB3 only	*N75*/*N326*	TM1	TM8
	K85/D311	**L1**	**L7**
7G8 only	K53/D57	JM1	JM1
	N58/E54	JM1	JM1
	R392/E54	TM10	JM1
	*SQ352/S72*	TM9	TM1
	T342/E232	**L8**	TM6
	N295/D313	**L7**	**L7**
Dd2 only	S157/E198	TM4	TM5
	R374/D377	**L9**	*TM10*
	R392/E399	TM10	*TM10*

a The column titled “hydrogen bond” indicates the participating residues, with the HB donor residue listed first. The columns titled residue 1 and residue 2 indicate location of the participating residues. Key HB are defined as ≤3.2 Å for >50% of simulation time across three independent 10 ns simulations (see text). For residues that are mutated in one or more isoforms, the relevant residue is italicized. An expanded table showing the lifetime of all HB for all isoforms found in ≥10% of simulation time for at least one isoform is found in the Supporting Information (Table S3).

The distance limit for significant, energetically favorable HB formation was defined as ≤3.2 Å between heteroatoms on amino acids capable of acting as HB donors and acceptors,^36^ and as in the case of SB (Figure S2), the HB summarized in Table S3 are found in ≥10% of the time across three independent simulations for at least one isoform. We summarize two types of HB, those present between two different amino acid side chains (Tables 3 and S3) and those present between a side chain and a neighboring peptide bond backbone (Tables 4 and S4); for the latter, the residue preceding the relevant backbone peptide bond is labeled in bold.

**Table 4:** Summary of Key Side Chain–Backbone Peptide Bond HB Found for 7G8^EMMD^, Dd2^EMMD^, and HB3^EMMD^ PfCRTs^a^

isoform	hydrogen bond	residue 1	residue 2
7G8, HB3, and Dd2	S65/**I61**	TM1	*TM1*
	S70**/I66**	TM1	TM1
	T82/**F78**	*TM1*	TM1
	T96/**V92**	TM2	*TM2*
	E95/**L254**	TM2	TM7
	Y109/**K116**	*TM2*	**L2**
	T149/**I146**	**L3**	*TM3*
	T151/**G147**	**L3**	*TM3*
	**G153**/E207	*TM4*	**L5**
	Q161/**C350**	TM4	TM9
	T193/**I189**	TM5	TM5
	S219/**L215**	TM6	TM6
	**T342**/E232	**L8**	TM6
	K236/**F340**	*TM6*	**L8**
	S250/**N246**	TM7	TM7
	S257/**Q253**	TM7	TM7
	**C312**/N295	**L7**	**L7**
	**T296**/D310	**L7**	**L7**
	S323/**F319**	TM8	TM8
	T333/**D329**	TM8	TM8
	S349/**Y345**	TM9	*TM9*
	Y384/**I347**	TM10	TM9
HB3 and 7G8	Q156/**T151**	TM4	**L3**
	**H180**/N183	*TM5*	*TM5*
	T230/**F226**	TM6	TM6
7G8 and Dd2	N167/**S163**	TM4	TM4
	*S220*/**L217**	TM6	TM6
	T265/**P262**	**L7**	*TM7*
HB3 only	S140/***A220***	TM3	TM6
7G8 only	*T76*/***S72***	TM1	TM1
	S134/**F130**	TM3	TM3
	N282/**SI279**	JM2	**L7**
Dd2 only	C171/**N167**	*TM4*	TM4
	*T356*/**Q352**	TM9	TM9

a In the column entitled hydrogen bond, the residue immediately preceding the relevant peptide backbone is in bold, and the HB donor residue is listed first. The columns titled residue 1 and residue 2 indicate location of the participating residues. Key HB are defined as ≤3.2 Å for >50% of simulation time across three 10 ns simulations (see text). Residues that are mutated across isoforms are italicized. An expanded table showing the lifetime of HB for all isoforms found in ≥10% of simulation time for at least one isoform is found in the Supporting Information (Table S4)

If an interaction involves a residue that is mutated in at least one other isoform, that residue is italicized.

**Figure 4:**
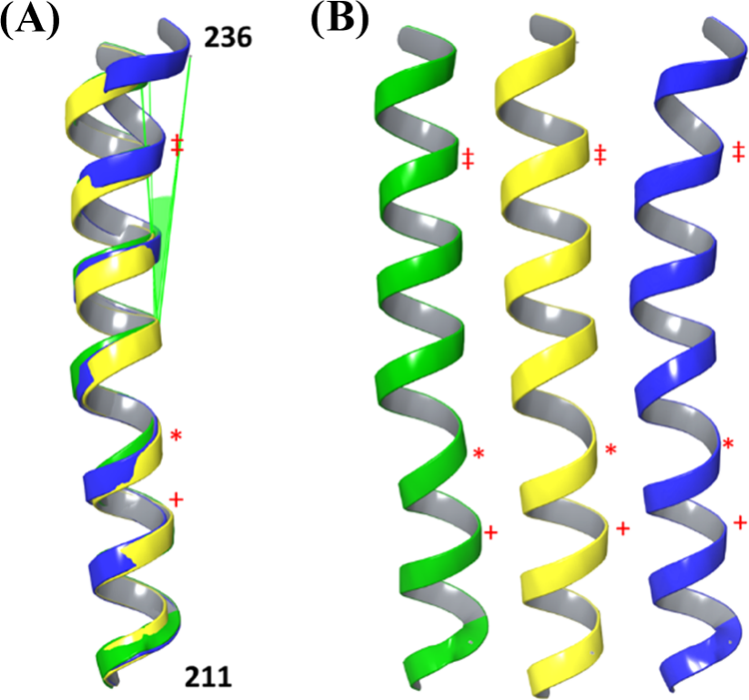
Illustration of the TM helix 6 bend that is correlated with the A220S substitution typically found in CQR isoforms (e.g., 7G8, Dd2). Structure of residues 211–236 was extracted from the corresponding EMMD structures (HB3 in blue, 7G8 in green, Dd2 in yellow). (A) Structures were superimposed after fixing the position of the protein backbone between residues 211 and 217. The angles that distinguish CQS from CQR PfCRTs were then measured as the angle between the α-carbons of residues 235 and 227 for 7G8 and Dd2 vs the α-carbon of residue 235 for HB3. The CQR isoforms deviate from HB3 by 16.4 and 13.5° for 7G8 and Dd2, respectively (green lines). Residue 220 (position of the common CQR-associated mutation), is indicated by the red asterisk, residue 217 is indicated by a red plus, and 231 is indicated by a red double dagger. The ends of the helices are defined by residues 211 and 236.^19^ The helix is oriented with the DV side of the membrane at the bottom. (B) Extracted TM6 structure for each isoform shown side by side to further highlight the different peptide backbone long axes; note the movement of the cytosolically disposed two helical turns for HB3 PfCRT (blue) relative to 7G8 and Dd2 PfCRTs (green, yellow, respectively).

The potential impact of all SB and HB is too extensive to summarize here; however, key differences that appear particularly relevant for drug binding are described in the next section. One interesting example of an important HB that does not appear to be directly involved in drug binding but that differs for CQS vs CQR PfCRTs is highlighted in Figure 4.

Although the enthalpic value of a single HB gained upon A to S substitution at position 220 in and of itself would not at first be expected to fully explain the TM6 structural change shown in Figure 4, we find that the common CQR-associated A220S substitution in PfCRT correlates with a ca. 4–16° bend within TM helix 6 near this residue (Figure 4), relative to HB3 PfCRT found in CQS parasites. This translates into predicted movement of the cytosolically disposed end of TM6 for CQR vs CQS isoforms (Figure 4). The bend correlates with loss of an S140 hydroxyl (donor)/A220 backbone peptide bond (acceptor) HB for HB3 PfCRT that changes to a longer-lived S220 hydroxyl (donor)/L217 backbone peptide (acceptor) HB for both CQR isoforms upon A220S mutation (Table 4), along with other isoform-specific interactions in this region indirectly related to A220S substitution. These include an E232/S341 HB that is longer lived for the CQR isoforms (Table S3). We suggest the cumulative effect of these is responsible for the TM 6 bend that distinguishes CQS from CQR PfCRTs (Figure 4).

**Figure 5:**
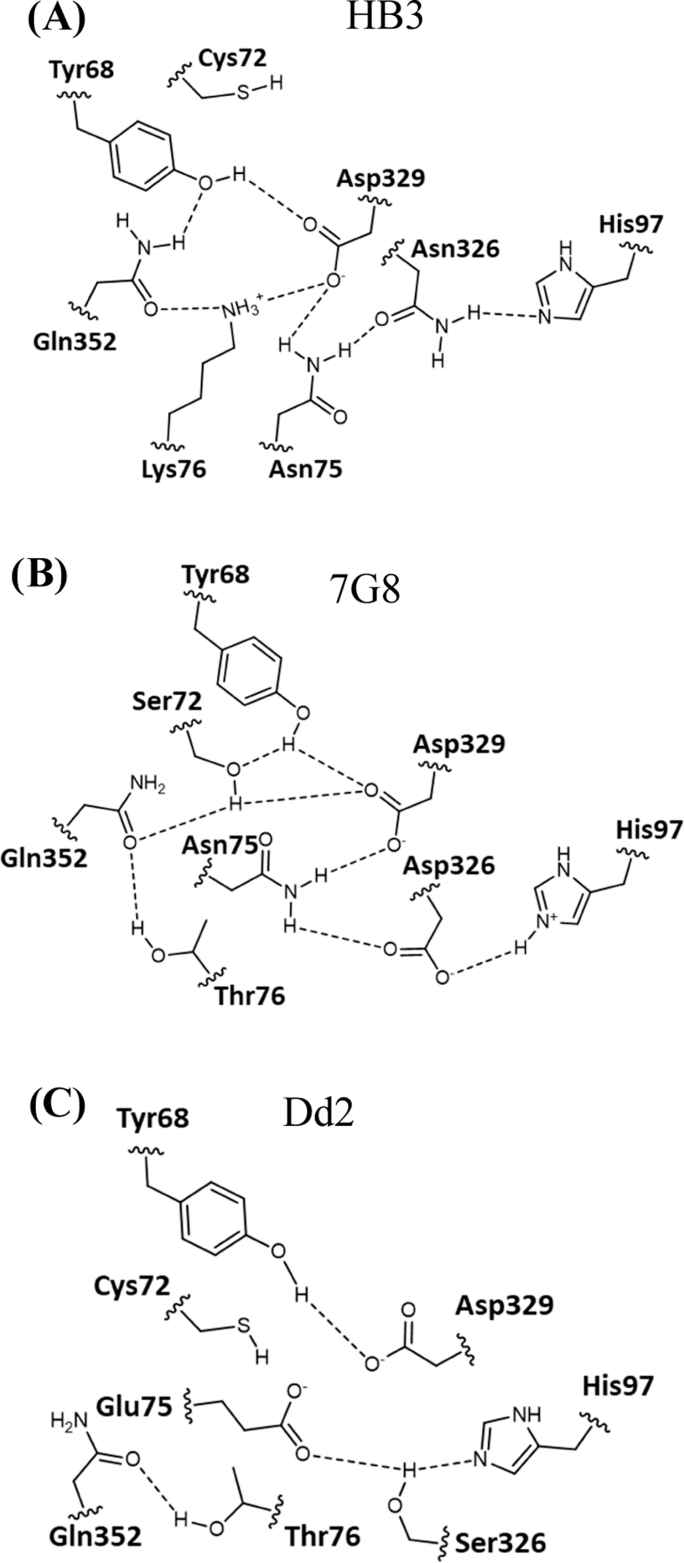
Network of salt bridges (SB) and hydrogen bonds (HB) surrounding residue 76 for the three different PfCRT isoforms studied here. We assign 6 HB and 1 SB for the HB3 isoform (A, top), and for the same residues, find 7 and 1 for the 7G8 isoform and 4 and 0 for the Dd2 isoform (B, middle; C, bottom), respectively.

As another key difference, we highlight the SB and HB “networks” near residue 76 that are found for the three isoforms (Figure 5).

These networks illuminate why CQR-associated *pfcrt* alleles that encode PfCRTs with sequences either S_72_VMNT_76_ (e.g., Dd2) or C_72_VIET_76_ (e.g., 7G8) relative to CQS (C_72_VMNK_76_) are particularly common, and highlight a previously unrecognized key role for D_329_ in PfCRT structure and function. For wild-type HB3 PfCRT, K at position 76 results in a unique network involving a trifurcated D_329_ interaction with residues at positions 68, 75, and 76, as well as coupled bifurcated N_326_/H_97_/N_75_ and Q_352_/K_76_/Y_68_ HB (Figure 5, top, “A”).

In contrast, mutation of K_76_ to T for the CQR isoforms, along with other mutations in this region (Table S1), results in rearranged isoform-specific networks (Figure 5B,C). For Dd2, loss of K_76_ along with mutation of N_75_ to E destroys the K_76_/D_329_ SB, rearranging the network near D_329_ to a longer-lived (cf. Table S3) Y_68_/D_329_ HB, with concomitant loss of the interaction between residues 75 and 329. Mutation of C_72_ to S for 7G8 PfCRT relative to the other two PfCRTs allows S_72_ to now form a trifurcated HB with D_329_, Y_68_, and Q_352_ and, similar to Dd2, loss of the K_76_ SB results in the formation of an HB between D_329_ and Y_68_. Thus, for both CQR isoforms, mutation of K_76_ to T_76_ disrupts multiple SB or HB that involve D_329_ in CQS-associated PfCRT. In contrast, although a different residue is present at position 326 for each of the three isoforms, formation of a bifurcated HB involving the 326 residue and H_97_ with either N_75_ (HB3, 7G8) or E_75_ (Dd2) is preserved in all three isoforms. We propose that relative energies and polarizabilities of the individual SB and HB in these isoform-specific networks help to explain differences in CQ binding and transport among the isoforms, particularly since drug binding involves some of the same network residues (described in the next section).

### Drug Docking

**Figure 6:**
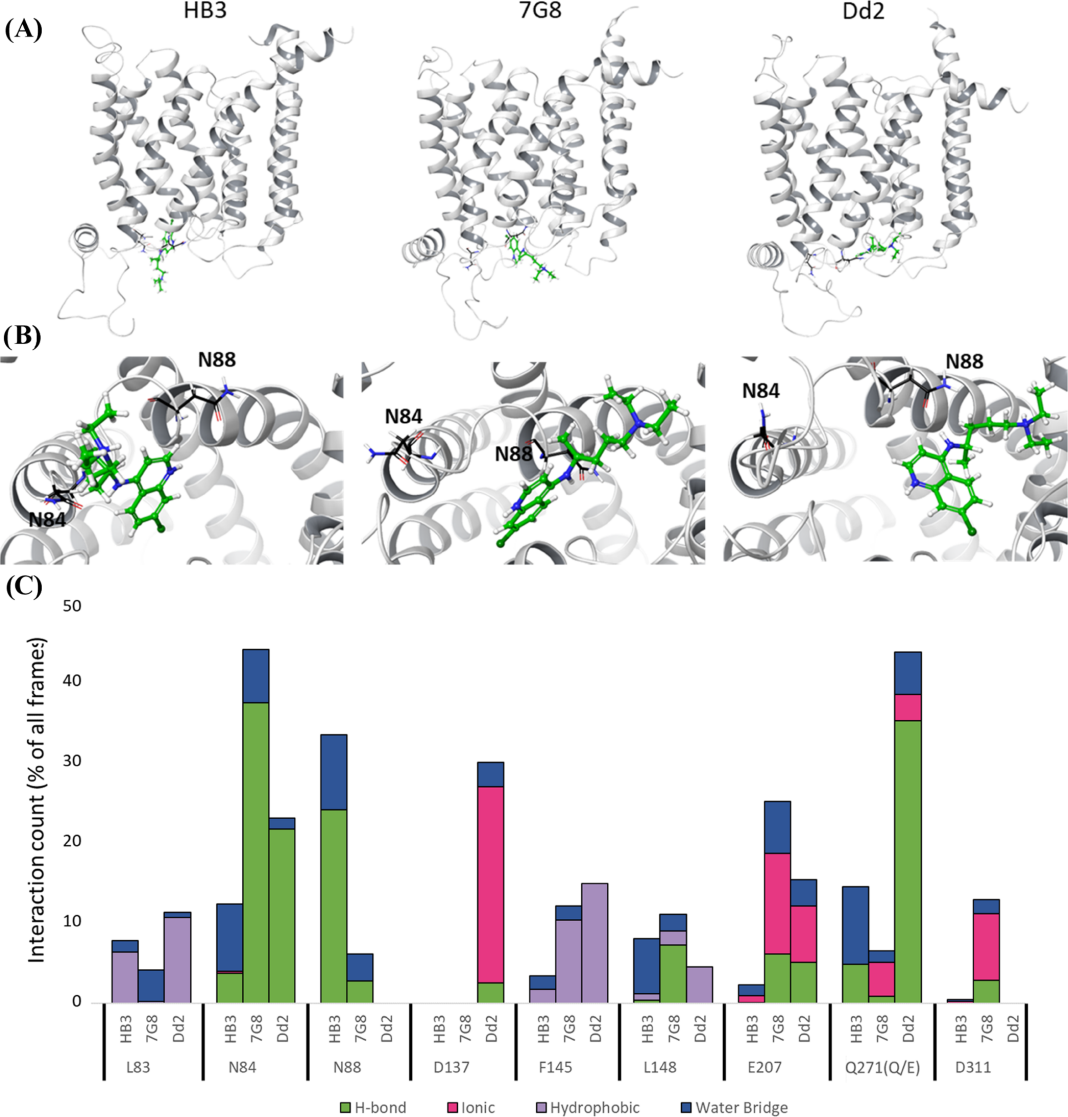
PfCRT side-chain interactions upon CQ binding to site A (see text). (A, top) Expanded drug binding site as seen from a side view of the protein isoforms. (B, middle) Close-up view of drug binding to site A, as seen looking from the DV disposed face “through” the PfCRT pore^19^ toward the cytosol. (C, bottom) Compiled MC/MD side-chain interaction data for CQ bound to site A for HB3, 7G8, and Dd2 PfCRT isoforms. When substituted in one or more isoforms, the HB3 residue is listed first (e.g., “Q271” denotes glutamine at position 271 for HB3 PfCRT) with the 7G8 and Dd2 residues listed in parentheses immediately following (e.g., “(Q/E)” after Q271, cf. Table S1). N84 and N88 interactions are identified as among the most long-lasting for 7G8, Dd2, and HB3 respectively. Each side-chain interaction is shown as a group of four types: HB (green), ionic (pink), hydrophobic (purple), and water-bridged SB or HB (blue). The value for each residue interaction is the average of three independent 100 ns simulations.

From initial docking of CQ^2+^ to isoform EMMD structures using SiteMap and Glide, followed by a second round of MC/MD to energy-minimize the drug–PfCRT complex after it is embedded in the membrane (see the Methods section), we visualize two drug binding sites (sites A, B) near the drug binding site previously experimentally defined by photoaffinity labeling and mass spectrometry under deenergized conditions.^17^ The two nearby sites show drug occupancy differences as defined by frame-by-frame analysis of side chain/drug interactions (below). The first defined drug binding site (A) is relatively long-lived, located at the DV opening of PfCRT, and overlaps quite closely with the Lekostaj CQ^2+^ binding site previously defined with a perfluoroazido CQ photoaffinity probe under deenergized conditions^17^ (Figure 6A,B).

**Figure 7:**
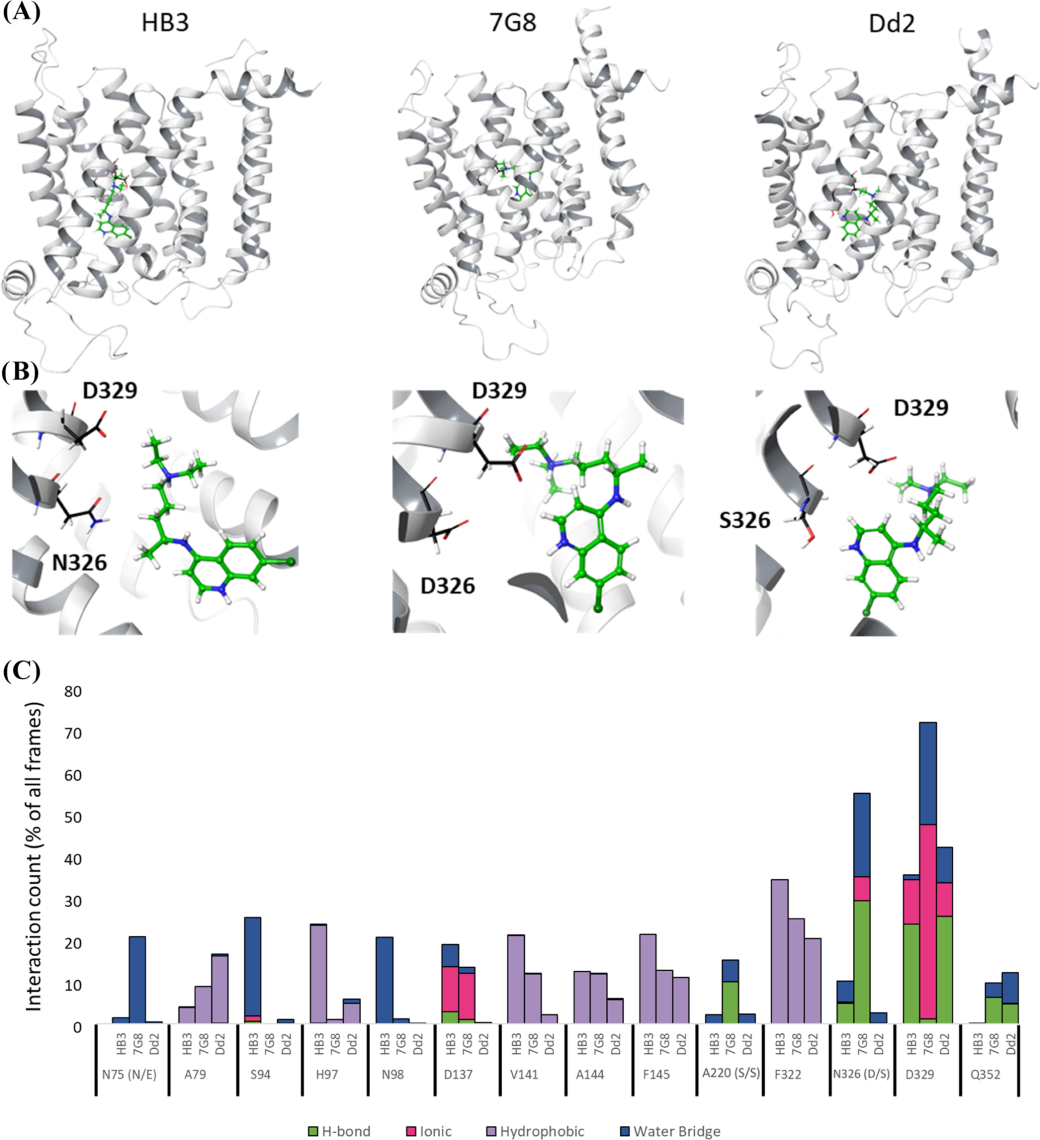
PfCRT side-chain interactions upon CQ binding to site B (see text). (A, top) Expanded drug binding site as seen from a side view of the protein isoforms. (B, middle) Close-up view of drug binding to site B, as seen looking from the DV disposed face through the PfCRT pore^19^ toward the cytosol. (C, bottom) Compiled MC/MD side-chain interaction data for CQ bound to site B for HB3, 7G8, and Dd2 PfCRT isoforms. When substituted in one or more isoforms, the HB3 residue is listed first (e.g., “N75” denotes asparagine at position 75 for HB3 PfCRT) with the 7G8 and Dd2 residues listed in parentheses immediately following (e.g., “(N/E)” after N75; cf. Table S1). Here, D329 is identified as an important residue for interaction with CQ, for all isoforms. Each side-chain interaction is shown as a group of four types: HB (green), ionic (pink), hydrophobic (purple), and water-bridged SB or HB (blue). The value for each residue is the average of three independent 100 ns simulations.

Site B is revealed only after MC/MD energy minimization of membrane-embedded EMMD structures bound to CQ^2+^, is located three to four helical turns further inside the central pore of the protein relative to site A, toward the cytosol, and lies proximal to the region 76 networks described above (Figure 7A,B).

We note that multiple transient substrate binding sites are often only revealed upon MC/MD energy minimization of experimentally derived transporter structures.^40^ As also suggested earlier,^19^ F′(ab) binding to PfCRT used to enable acquisition of 7G8^EM^ structure presumably “locks” PfCRT into one “open” conformation such that site B is only revealed after MC/MD energy minimization of the drug–PfCRT complex. Site B is presumably not accessible in the PfCRT “closed” state, but only in an open state for membrane-embedded PfCRT as energy-minimized here or as transiently formed under physiologic conditions in the presence of DV membrane ΔpH and/or ΔΨ (see the Discussion section).

The geometry of CQ^2+^ docked within site A (Figure 6) is entirely consistent with previous photoaffinity labeling that places CQ^2+^ proximal to loop 9.^17^ The geometry of CQ^2+^ docked within site B (Figure 7) has not previously been suggested but in a general way has been hypothesized for quite some time since it is proximal to the K_76_ residue found in wild-type (CQS-associated) PfCRT that is then mutated to neutral T for all known CQR-associated PfCRTs.^1,12,41^ It has long been suspected that loss of positively charged K_76_ for most PfCRTs found in CQR strains permits better access to the transporter pore interior for positively charged drugs such as CQ^2+^ during their transport from the parasite DV to the cytosol.^41^ However, based on the geometry of CQ docked at site B revealed here (Figure 7), as well as the above definition of the region 76 SB/HB network (Figure 5), we suggest that CQR isoform-associated “release” of D_329_, upon mutation of K_76_ in CQR-associated PfCRT isoforms, which would otherwise participate in a unique, more stable D_329_/K_76_/N_75_/Y_68_ interaction as found for CQS-associated PfCRT (Figure 5A) more precisely explains improved access of CQ^2+^ to site B for CQR isoforms since stable interaction between negative D_329_ and positive CQ^2+^ would then become more frequent for CQR isoforms of PfCRT as we now find (Figure 7B,C).

For CQ bound within site A, residues N_84_ (Dd2, 7G8) or N_88_ (HB3) are predicted to be particularly prominent in stabilizing initial drug binding for CQR vs CQS isoforms (Figure 6). For example, for 7G8^EMMD^, a CQ-N_84_ HB is present during >40% of the frames in the clustered three MC/MD simulations, for Dd2, it is present during ∼1/4 of the simulation, but for HB3, it is present <15% of the time with the majority of that time as a weak water-bridged interaction, whereas much stronger interaction with N_88_ occurs during ∼1/3 of simulation time for HB3 and little to none of the time for the 7G8 and Dd2 isoforms (Figure 6C). That is, N_84_ interaction is more stable for Dd2 and 7G8, and appears almost exclusively HB in character, vs a less frequent and 1:2 ratio of HB/water-bridge interactions found for HB3, which results in overall weaker interaction with the residue. The converse appears to be the case for N_88_ (Figure 6C). This suggests an N_84_ carbonyl O/CQ quinolinal N interaction is relevant for initial binding of drug to CQR-associated PfCRT, whereas N_88_ is more important for initial drug binding to HB3 PfCRT.^17^ Other residues that appear to distinguish site A drug interaction for CQR vs CQS isoforms include D_137_, E or Q_271_, and E_207_ (Figure 6). Through MC/MD frame counting, the E_271_ interaction that is specific for Dd2 appears to be significantly more stable, compared to the Q_271_ interaction for HB3 and 7G8 (Figure 6C). These isoform-specific PfCRT residue/drug interactions likely explain, at least in part, the pH-dependent differences in initial CQ binding affinity measured for CQR vs CQS isoforms under deenergized conditions.^2,17,19^

Within Site B, side-chain interaction differences are again observed across the different PfCRT isoforms (Figure 7). Both CQR isoforms interact more strongly with CQ^2+^ via D_329_ (Figure 7C), a residue that as noted above forms a strong SB with K_76_ in the HB3 isoform in the absence of drug and CQR-associated K76T substitution (Figure 5). For 7G8, the CQ^2+^–D_329_ interaction appears to alternately involve both the tertiary aliphatic and quinolinol N of CQ^2+^ (not shown), appears primarily ionic in nature, and persists for ∼70% of simulation time (Figure 7C). In contrast, for Dd2, the D329 interaction appears more HB in nature, primarily involves the CQ^2+^ quinolinol N (not shown), and persists for >40% of simulation time (Figure 7C). These interesting differences are a consequence of the unique region 76 SB/HB network rearrangements defined above (cf. Figure 5 vs 7B,C).

A D_329_–CQ^2+^ interaction also has reduced, but easily measurable, lifetime in the CQS-associated HB3 isoform, but only exists for ∼35% of simulation time (Figure 7C), consistent with HB3 PfCRT binding and transporting CQ at lower but measurable efficiency relative to 7G8 and Dd2 PfCRTs.^2,4^ Since K_76_ is present in the correct position and geometry to form a D_329_–K_76_ salt bridge for HB3 PfCRT (Figure 5), we suggest that K_76_ is not directly involved in binding drug, but competes with CQ^2+^ for ionic interaction with D_329_ in HB3 PfCRT.

### AlphaFold Structures

**Figure 8:**
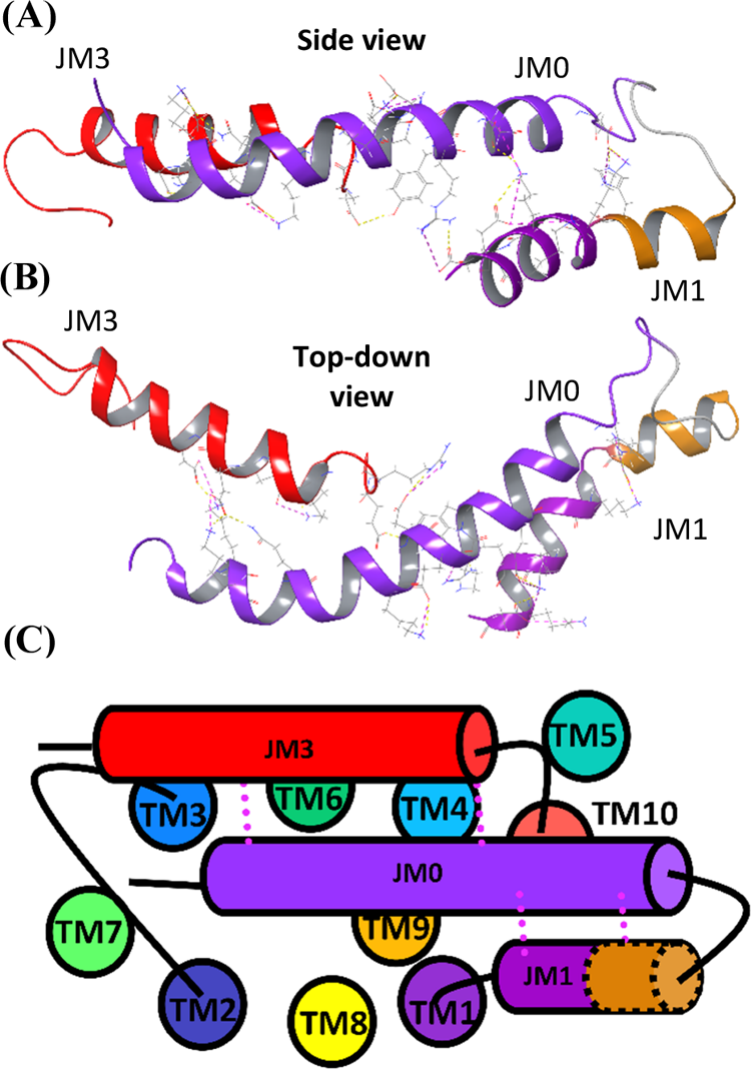
Side (A, top), top down (B, middle), and cartoon top down (C, bottom) views of the AFMD energy-minimized PfCRT 3-helix zipper for HB3^AFMD^. These structural elements arise from the AF2-resolved C terminus (residues 400–424) and N-terminus (residues 1–33) folding together after energy minimization for the membrane-embedded HB3^AF^ structure, along with folding vs newly resolved JM1 residues 40–56 and the previously resolved JM1 segment^19^ (see text). The zipper is stabilized by a large number of SB ((A, B) purple dotted lines) and HB ((A, B) yellow dotted) that will be discussed elsewhere. JM0, JM1, and JM3 segments are shown in purple, dark purple/orange, and red, respectively.

Recently, the second-generation AlphaFold Artificial Intelligence (AFAI) algorithm “AF2” has been shown to quite accurately predict atomic-resolution three-dimensional protein structures from the primary amino acid sequence, including in the case of CRT proteins.^21,32,38^ As mentioned, for the 7G8^EM^ PfCRT structure, residues 1–46 (much of the N terminus), 406–424 (much of the C terminus), and 112–124 (cytosolically disposed L2 between helices 2 and 3) are unresolved, thus structure for these segments is missing from the pdb file deposited for 7G8^EM^ (# 6UKJ) and hence from the energy-minimized EMMD structures solved for the three isoforms using 7G8^EM^ as the initial template (Figure 1). Previously published 7G8^EM^ structure images omit the N and C termini regions altogether and denote structurally unresolved L2 residues with a dashed line.^19^ We therefore increased our computational capabilities to apply AF2 software and test whether these segments influenced the energy-minimized EMMD structures solved above. PfCRT structure has previously been determined for the wild-type (HB3) isoform using AF2^38^ but not for the CQR conferring isoforms, and no previous AF2-generated structure has to our knowledge been embedded in the membrane and further resolved by MC/MD energy minimization. As shown, including these segments and solving for energy-minimized PfCRT via tandem AF2 followed by MC/MD simulation yields 10 TM core PfCRT structure that is similar to the static structure solved by cryo-EM (Figures 3A and S4). However, as noted above, additional structure for the regions unresolved by cryo-EM for 7G8^EM^ is also revealed by AF2, and is similar to that previously found for AF2-generated structures of *Plasmodium berghei*, *Plasmodium vivax*, *Plasmodium chabaudi*, and *Plasmodium knowlesi* CRT protein orthologues.^38^ This includes an ∼5 helical turn C terminal helix extension that prior to energy minimization appears to extend out of and perpendicular to the membrane surface plane, as well as an extended N terminus that forms two helical segments that fold back upon each other (Figure 3B, green). The AF2 extended N terminus includes an additional two helical turns that lengthens the previously defined 7G8^EM^ “JM1” helix region,^19^ followed by a short loop (residues 34–38) and another helical segment (residues 1–33) that we now denote “JM0” (Figures 8 and S7). When HB3^AF^ is energy-minimized (to solve for HB3^AFMD^) and HB3^AFMD^ is superimposed upon energy-minimized HB3^EMMD^ (Figure 3A), the core 10 TM PfCRT structures are again very highly conserved (Figure 3A caption; all-atom RMSD = 2.86 Å) in spite of the presence of these newly resolved L2 and N and C termini regions. These results are remarkable and further validate the energy-minimized isoform EMMD structures as well as routine application of AF2.

Starting with HB3^AF^ but otherwise using the same procedure that generates HB3^EMMD^ from HB3^EM^, and averaging three independent MC/MD simulations yields energy-minimized HB3^AFMD^ structure (Figures 3A,C and 8). Not surprisingly, energy minimization to solve for HB3^AFMD^ results in the C terminal helical extension (we call this “JM3”; purple, Figures 8 and S7) bent downward to more stably align with the membrane surface (compare green regions, Figure 3C vs 3B and Figure 8).

Upon doing so, the C terminal JM3 helical segment now interacts with the N terminal 4 turns of JM0, while the C terminal 3 turns of JM0 simultaneously pair with the N terminal 3 turns of JM1 (Figures 8 and S7). Interestingly after MC/MD energy minimization, the three-helix bundle appears to fold directly above the cytosolic opening of the PfCRT central pore (Figure 8C). The three-helix bundle is stabilized by numerous SB and HB generated by acidic, basic, and polar residues positioned along the three helical segments in a precise amphipathic helical geometry (Figure 8A,B) that will be described in more detail elsewhere. Of note, many of the residues forming this intrahelical stabilizing SB/HB network, as well as other SB/HB described above are exceedingly well conserved across at least 24 CRT orthologues from a number of species^42^ (Figure S8).

We note that small three-helix bundle structures are found in many proteins including villin and a number of DNA binding proteins,^43,44^ but the helical segments are often shorter, contiguous, and arranged with one perpendicular to the other two. Since in the case of PfCRT the participating helical segments are dis-contiguous and are mutually parallel, we name this helical bundle the “3-helix zipper”. The zipper appears to fold directly above the cytoplasmic facial opening of the PfCRT central pore (Figures 8 and S7) perhaps suggesting a novel gating mechanism for the release of CQ^2+^ from the central pore into the parasite cytosol during the drug transport cycle.^45−47^

**Figure 9:**
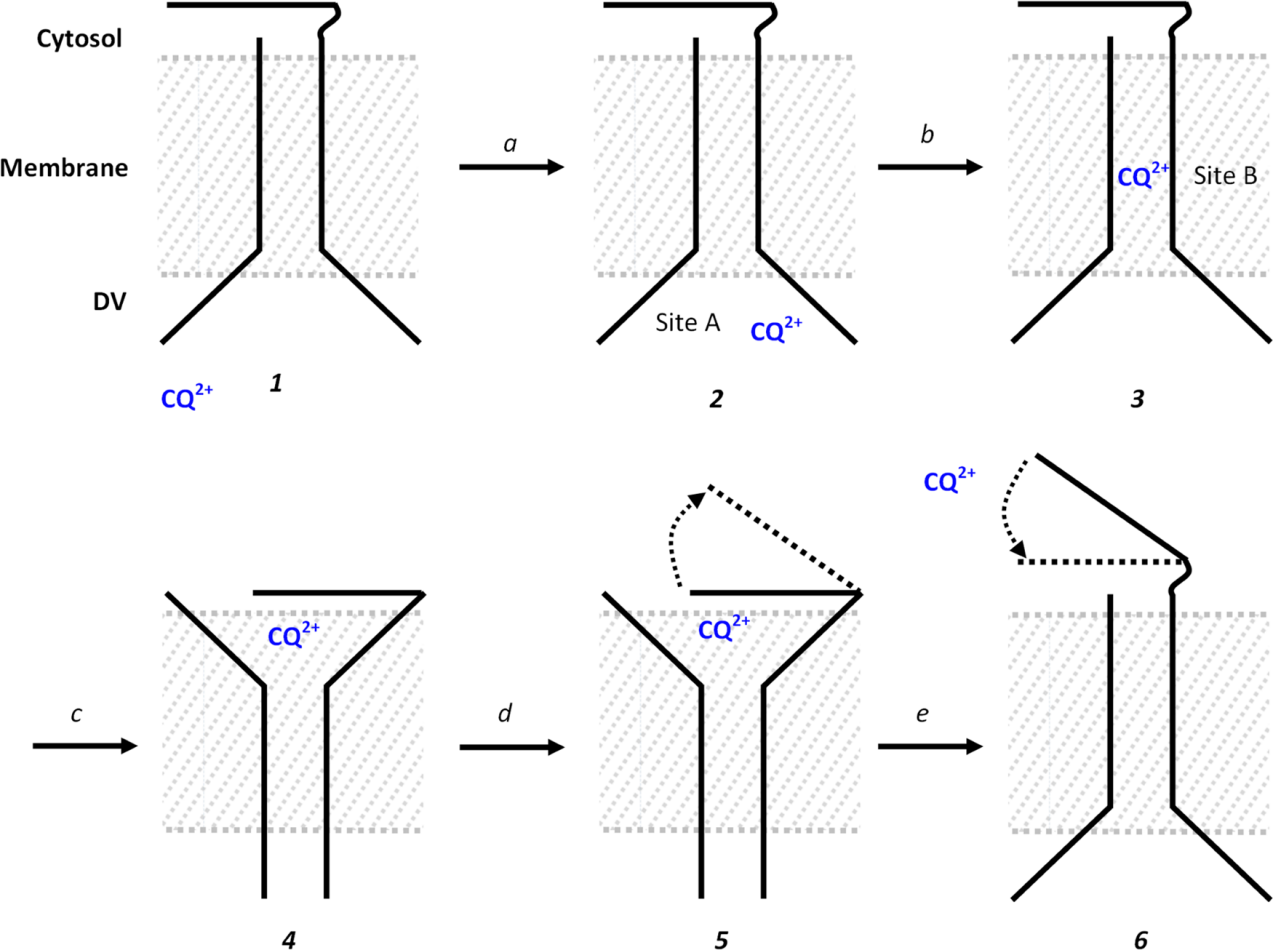
Proposed drug transport model. (1) PfCRT in open conformation before drug association. (2) CQ^2+^ binds to site A including via residues N84 or N88 in step “a”. (3) CQ is translocated to site B in step “b”. (4) CQ^2+^ is translocated to near the cytosolically disposed 3-helix zipper (cf. Figure 8) in step “c”. (5) ΔpH- or ΔΨ-dependent conformational change “opens” the cytosolically disposed zipper in step “d” (solid horizontal to dashed line). (6) CQ is released, and the protein returns to the “open to DV” conformation during step “e”.

Finally, using these structural insights, we propose a simplified model for CQ transport from the *P. falciparum* DV to the parasite cytosol via PfCRT (Figure 9). This model includes novel features revealed in the above and is consistent with virtually all data so far collected for PfCRT. The model incorporates two transient drug binding sites (“A” and “B” as described above) as well as a conformationally active three-helix bundle “zipper” (described above, Figure 8) that may be involved in gating the release of drug at the cytosolic face of the PfCRT central pore.

As previously determined using a CQ photoaffinity probe and mass spectrometry “footprinting”,^17^ we propose initial binding of CQ^2+^ to the DV disposed “site A” (Figure 9, “2”). Our previous definition of site A^17^ was done under deenergized conditions wherein PfCRT does not experience a transmembraneous ΔpH or ΔΨ and is not locked into an open state by F′(ab) binding as is the case for the 7G8^EM^ PfCRT structure.^19^ Thus, not surprisingly, MC/MD of locked 7G8^EM^ to yield 7G8^EMMD^ followed by CQ^2+^ docking and a second round of MC/MD reveals site A as well as a second but shorter-lived “site B” that involves interaction with different residue side chains (“3”; Figure 7). We propose that ΔpH- or Δ Ψ-driven translocation from “A” to nearby “B” (Figure 9, step b) is facilitated by concomitant effects on the protonation state of CQ-coordinating residues and rearrangement of region 76 SB/HB networks (Figure 5) as elaborated upon further in the Discussion section. Release of CQ^2+^ to the cytosol may be mediated in part by the three helical segment zipper at the cytosolic face of PfCRT (steps c–e) that we identify after MC/MD energy minimization of AF2 structures generated for membrane-embedded PfCRT. Higher DV transmembrane ΔpH experienced by CQR-associated PfCRTs due to lower DV pH for CQR parasites^48−51^ is envisioned to increase the rate of steps b–e for CQR parasites via effects on polarized SB/HB^52^ as described above for the region 76 network.

## Discussion

Initial definition of a single CQ drug binding site for PfCRT was done with a CQ photoaffinity probe and membrane-bound HB3, Dd2, and 7G8 PfCRTs under deenergized conditions,^17^ whereas experimental definition of PfCRT atomic-level stucture was done by cryo-EM for purified 7G8 PfCRT protein “locked” into one conformation by an attached F′(ab) antibody fragment.^19^ Here, we perform detailed MC/MD energy minimization calculations for PfCRT isoforms embedded in membrane with or without CQ^2+^ bound starting with locked 7G8^EM^ as the initial template. We stress that these three approaches are not necessarily expected to yield identical results; nonetheless, they provide key conclusions that are remarkably consistent. By combining analyses of these three data sets, as well as additional data published by others,^45,53,54^ we are able to draw several important conclusions that we test further using MC/MD energy-minimized structures of full-length PfCRT generated by AFAI:

(1) MC/MD energy minimization of 7G8^EM^ after embedding in membrane yields a core 10 TM helical structure for 7G8^EMMD^ that is quite similar to the structure solved by cryo-EM,^19^ and substitution of amino acids that distinguish HB3 and Dd2 PfCRT isoforms from 7G8 PfCRT, followed by MC/MD energy minimization of these isoforms embedded within a membrane, again yields quite similar 10 TM domain core structures, but with small local structural perturbations. These perturbations are due at least in part due to the disruption or formation of key SB and HB directly or indirectly due to the amino acid differences that characterize the isoforms. We propose that these SB/HBit rearrangements are responsible for the differences in CQ binding and transport that have been measured to date for these PfCRTs.^2,4,12,17^
(2) Core 10 TM domain membrane-embedded AFMD structures generated after tandem AFAI and MC/MD energy minimization are quite similar to those found for the corresponding EMMD PfCRT isoform structures. We note that the use of post-prediction MD has previously been utilized to compensate for gaps that are present in a static structure solved with diffraction methods.^55^ The tandem AFAI MC/MD approach used here reveals structural details that were not previously resolved by cryo-EM. These include a cytosolically disposed three helical segment zipper that could be involved in gating the cytosolic side of the PfCRT drug pore. More detailed analysis exploring this point will be presented elsewhere.
(3) Considering all data and conclusions from the three approaches, as well as other observations^18,45,53,54^ allows us to propose a simplified model for PfCRT drug transport under physiological conditions. In this model, we invoke two dynamic proximal drug binding sites (A, B; Figure 9) that are found for all isoforms after MC/MD energy minimization of membrane-embedded PfCRTs.

Previously, comparison between CRT proteins expressed in multiple *Plasmodia* spp. and atomic-resolution structures for other DMT family members allowed Coppée et al.^42^ to construct a non-energy-minimized homology model for PfCRT that is similar to the recently solved 10 TM core 7G8^EM^ structure; however, this homology model did not include extended N and C termini regions defined here by AFAI and is not amenable to analysis of small local structural differences among isoforms. AFAI-generated structures described here and solved previously^38^ show remarkably similar 10 TM core PfCRT structure and in addition reveal important loop and termini domain structure not found for 7G8^EM^ or the Coppée homology model.

Our MC/MD calculations suggest that along with known amino acid substitutions and the local structural perturbations that they promote, conformations of JM1, the cytosolically disposed end of TM 6, and the PfCRT cytosolic tail (JM3) distinguish CQR from CQS PfCRTs. Also, although not shown, the DV ends of TMs 3–6 for the HB3 CQS-associated isoform appear to be pulled slightly closer into the center of the pore and along with the A220S substitution-induced TM6 bend described for CQR PfCRTs may alter the drug pore openings for CQR-associated PfCRTs (Figure 4).

There are 17 SB common to the three PfCRT isoforms studied here (Table S2); 19/39 residues involved are TM residues and 11 are strictly conserved across 24 orthologous *Plasmodium* CRT sequences from a variety of species,^42^ with the remainder being highly conserved (Figure S8).

High sequence conservation supports the conclusion that these SB support endogenous PfCRT and CRT orthologue protein function.^19^ In contrast, there are eight SB unique to either the 7G8 or Dd2 CQR conferring PfCRT isoforms, these may contribute to increased CQ transport by CQR PfCRT isoforms in an isoform-specific fashion. We also identify four SB unique to HB3 PfCRT (Table S2). Two of these involve one helix and one loop residue. These might also influence drug transport by affecting loop flexibility and/or sequestering negatively charged residues, preventing them from otherwise interacting with protonated drug.

A particularly important SB is K_76_/D_329_. As mentioned, K76T substitution is particularly common in CQR conferring PfCRT isoforms and loss of K-associated charge has been suspected for some time to be related to different CQR vs CQS isoform function.^12,41^ Here, we extend and enhance our understanding by finding that K_76_–D_329_ distance across multiple independent MC/MD simulations for HB3 PfCRT indicates the bridge is conformationally active and that K_76_ is involved in an extended SB/HB network that is unique for CQS-associated PfCRT. The importance of this SB is further illuminated by transient interactions between D_329_ and CQ^2+^ during drug docking simulations (Figure 7). K76T mutation obviously prevents the formation of a K_76_/D_329_ SB for the CQR isoforms; we suggest that breaking this endogenous SB leads to more frequent CQ^2+^ binding to site B and increased drug transport by allowing D_329_ to interact more frequently with CQ^2+^ (Figure 5). Further analysis of second-site revertants^12^ with T at position 76 but that also transport CQ^2+^ better than HB3 PfCRT should prove informative, as would conservative substitution of these residues and measurement of resultant effects on drug transport.

Where an isoform-specific SB is broken (e.g., K_76_/D_329_ broken for 7G8 and Dd2), we can suggest possibilities for how the liberated charge is stabilized. Some bridges are broken due to mutation to an uncharged residue subsequently unable to form an SB but an alternate HB is formed instead; e.g., the K_76_/D_329_ SB is only present in HB3 PfCRT, but T_76_/Q_352_ and stronger D_329_/Y_68_ HB are found for 7G8 and Dd2 PfCRTs. In other cases, compensating counterions form alternate SB (e.g., while bifurcated R_392_/E_399_/R_404_ is only present in HB3 (Table S2), E_399_/K_402_ is present in Dd2, where K_402_ appears to replace R_392_ and R_404_). In these scenarios and others, the concomitant rearrangements in protein structure promote additional small conformational changes that presumably impact function.

We identify 12 strong side chain–side chain HB common to all three isoforms (green, Table S3). 17/19 of participating residues involved lie within helices, and their HB show long lifetimes during MC/MD, suggesting they are quite stable. Again, interestingly, 15/19 of these residues are strictly conserved across 24 CRT orthologues (Figure S8), with three others identical in 23 of 24 orthologues,^42^ supporting the notion that these HB are relevant for endogenous CRT protein function. We find 11 side chain–side chain HB unique to 7G8 and Dd2 (Table 3). These perhaps contribute to enhanced drug transport in an isoform-specific manner. Alternatively, two side chain–side chain HB are unique to HB3 PfCRT expressed in CQS parasites: N_75_/N_326_ and K_85_/D_311_. The first involves residues within helices (TM1, TM8) with the second involving two loop residues (L1, L7). Finally, we emphasize H_97_/N(or E)_75_/N (or D or S)_326_ as an important interaction since it is found for all isoforms with H_97_ involved in forming either an HB or SB within the 76 region network, depending on the isoform, and because H97Y has recently appeared as an additional substitution associated with drug resistance.^10,56^ How H97Y substitution perturbs the 76 region interactions identified here is worthy of additional study. We note that Ross et al. suggest that the H97Y substitution may affect resistance to both CQ and piperaquine (PPQ);^10^ thus, it is possible that the substitution affects binding of quinoline-based drugs to site B. Indeed, Ndung’u et al. note that an H to P substitution at the analogous position for *P. berghei* PbCRT promotes increased resistance to amodiaquine (AQ).^57^ We recently found that H97Y substitution within Dd2 PfCRT alters PPQ and CQ transport in a manner consistent with the interpretation offered by Ross et al.;^10^ however, an anticipated correlative reciprocal relationship for transport of these two drugs across a collection of PPQ resistance-conferring PfCRT mutants was not found.^11^ Interestingly, another residue that is involved in the region 76 SB/HB network for all isoforms is Q352, which we note may mediate increased resistance to yet another quinoline-based antimalarial drug, quinine, when the residue is changed to either K or R.^58^ These points, and additional, merit detailed study by the methods shown here, perhaps in combination with analysis of PfCRT site-directed mutants.

Similar analysis can be performed on side chain–peptide backbone HB shown in Tables 4 and S4, but given their large number, only a few are highlighted here. E_95_/**L**_**254**_, K_236_/**F**_**340**_, and **C**_**312**_/N_295_ are HB found in all three isoforms. Residues E_95_, F_340_, and C_312_ are strictly conserved across 24 CRT orthologues (Figure S8), with K_236_ and N_295_ very highly conserved (23/24 and 22/24, respectively) and L_254_ existing only as homologous L, I, or F in the other orthologues.^42^

The novel three-helix zipper structure that we find for PfCRT upon tandem application of AFAI and MC/MD is quite interesting. As mentioned, three-helix bundles are common in proteins but often the residues within the three segments are contiguous and one helical segment is typically oriented perpendicular to the other two. Here, the residues involved are dis-contiguous and the segments are mutually parallel. Naturally occurring three-helix bundles bind a number of substrates^44^ and they have also been engineered to readily bind small-molecule ligands.^59^ We note that regulatory short helical bundles oriented parallel to the membrane, reminiscent of the PfCRT zipper, have been found for other transporters including the HCN nucleotide channel, as well as Shaw Kv and Vibrio ion channels.^60−63^

Although the physiologic function of PfCRT is currently debated, function is likely essential since no successful *pfcrt* knockout has yet been reported. Possible physiologic substrates for PfCRT that have been suggested include ions, amino acids, glutathione, and other peptides.^2,19,49,50,64^ These merit further study by the methods described here.

Finally, we note small but interesting pH-dependent differences in the affinity of drug binding to various preparations of CQS vs CQR isoforms of PfCRT reported previously vs what is observed in this study. Initial reports of Zhang et al.^2^ and Lekostaj et al.^17^ found small differences in CQ binding affinity for CQS vs CQR PfCRT isoforms expressed in yeast plasma membrane measured at pH 6.5 and at a range of pH 5.0–8.0, respectively. Although binding measurements in Lekostaj et al. were via photolabeling with a CQ probe, wherein equilibrium affinity cannot be calculated, lower pH was found to be consistent with higher-affinity binding. Similarly, although Kim et al.^19^ found little difference in CQ binding affinity at pH 7.5 for nanodisk preparations of CQS vs CQR PfCRT isoforms, small differences similar to those seen by Zhang et al.^2^ were found at pH 5.5. Although some difference in drug affinity for membrane vs nanodisk preparations of PfCRT might be expected, and although more study via the methods reported here is certainly needed, the weak base diprotic nature of CQ and side-chain interactions that we find here for CQ^2+^-docked to energy-minimized PfCRT isoform structures are consistent with pH-dependent and isoform-specific effects on drug binding similar to those reported to date.

## Conclusions

In summary, we have shown that along with known amino acid substitutions and the local structural perturbations that they promote, conformations of PfCRT regions JM1, the cytosolically disposed end of TM 6, and a newly described segment of the PfCRT cytosolic tail (JM3) distinguish CQR from CQS PfCRT isoforms. Future studies will include additional analyses of “region 76” residue networks as well as the functional role(s) of the newly identified PfCRT zipper. Both additional site-directed mutagenesis experiments and MC/MD energy minimization as described here will be valuable.

## Supporting Information Available

Figures showing computational workflow, colored RMSD structures, and heatmaps of interactions, and tables containing lifetimes of all SB and HB interactions (PDF)

## Accession Codes

PfCRT (HB3 or 3D7, UniProt): Q8IBZ9. PfCRT (7G8, UniProt): W7ET94. PfCRT (Dd2, UniProt): D5L5S1. PfCRT (7G8, PDB): 6UKJ.

## Abbreviations

**AFAI**: AlphaFold artificial intelligence
**AF2**: AlphaFold2 algorithm
**AFMD**: energy minimized using AF2 structure template
**AQ**: amodiaquine
**CQ**: chloroquine
**CQR**: chloroquine resistant/resistance
**CQS**: chloroquine sensitive
**DV**: digestive vacuole
**EMMD**: energy minimized using cryo-EM structure template
**HB**: hydrogen bond
**IC**_**50**_: 50% optimal growth inhibitory dose
**JM**: juxta(posed) (to) membrane
**LD**_**50**_: 50% optimal lethal dose
**MC/MD**: Monte Carlo molecular dynamics
**PfCRT**: *Plasmopdium falciparum* chloroquine resistance transporter
**PK/PD**: pharmacokinetics/pharmacodynamics
**POPC**: 1-palmitoyl-2-oleoyl-*sn*-glycero-3-phosphocholine
**PPQ**: piperaquine
**RMSD**: root mean square deviation of atomic positions
**SA**: South America
**SB**: salt bridge
**SEA**: South East Asia
**SPC**: simple point charge
**TB**: terabyte
**TM**: transmembrane
