# Supplementary material for: Structures of Plasmodium falciparum Chloroquine Resistance Transporter (PfCRT) Isoforms and Their Interactions with Chloroquine
Source: Biochemistry. 2023 Feb 17;62(5):1093–110. doi: 10.1021/acs.biochem.2c00669 (PMC10950298; doi:10.1021/acs.biochem.2c00669)
Supplement: Supplementary file 1 — bi2c00669_si_001.pdf [file bi2c00669_si_001.pdf]

Supplemental Information
for
"STRUCTURES OF *PLASMODIUM FALCIPARUM* CHLOROQUINE RESISTANCE TRANSPORTER (PfCRT) ISOFORMS AND THEIR INTERACTIONS WITH CHLOROQUINE"

Andreas Willems [#], Adrian Kalaw [#], Ayse Ecer, Amitesh Kotwal, Luke D. Roepe[+] & Paul D. Roepe [*]

Depts. of Chemistry and of Biochemistry and Cellular and Molecular Biology,
Georgetown University
37th and O Streets NW
Washington DC 20057

1. Figures S1 – S8 with attached captions.

2. Tables S1 – S5 with attached captions.

3. List of reported EMMD and AFMD pdb files available from the authors.

SUPPLEMENTAL FIGURES

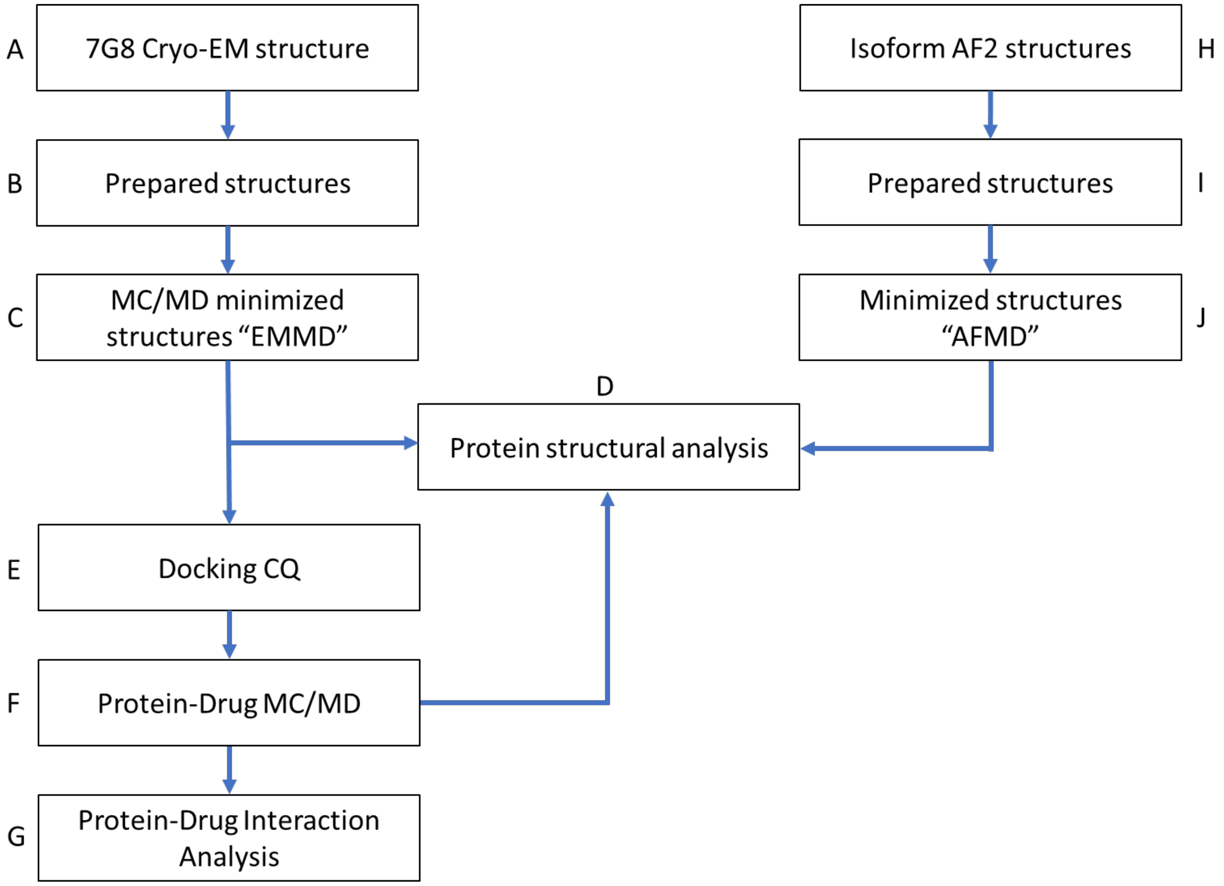

Figure S1. Flowchart of computational work. **A**) The 7G8^{EM} (PBD 6UKJ) structure is imported into Maestro. **B**) Mutations are made *in silico* to generate additional HB3 and Dd2 isoform primary sequence, and protein structure for all membrane imbedded isoforms is prepared for MC/MD energy minimization (see methods). **C**) Energy minimized structures are generated for all isoforms by MC/MD and all frames from three independent simulations are clustered. **D**) The averaged structures are analyzed and salt bridges (SB) and hydrogen bonds (HB) are inventoried as described in methods. **E**) Structures are docked to CQ^{2+}. **F**) The two highest ranked (see "drug docking", Methods) drug-poses are energy minimized as in **B,C.** **G**) docked structures are analyzed for protein-drug interactions (see text). **H**) AF2 structures are generated for HB3, 7G8, and Dd2 PfCRT isoform using isoform primary sequence. **I**) The AlphaFold (AF2) structures are membrane imbedded and prepared for MC/MD energy minimization as in "**B**" (see methods). **J**) The AF2 energy minimized structures are solved by MC/MD (as in "C") and all frames clustered to generate the presented "AFMD" structures.

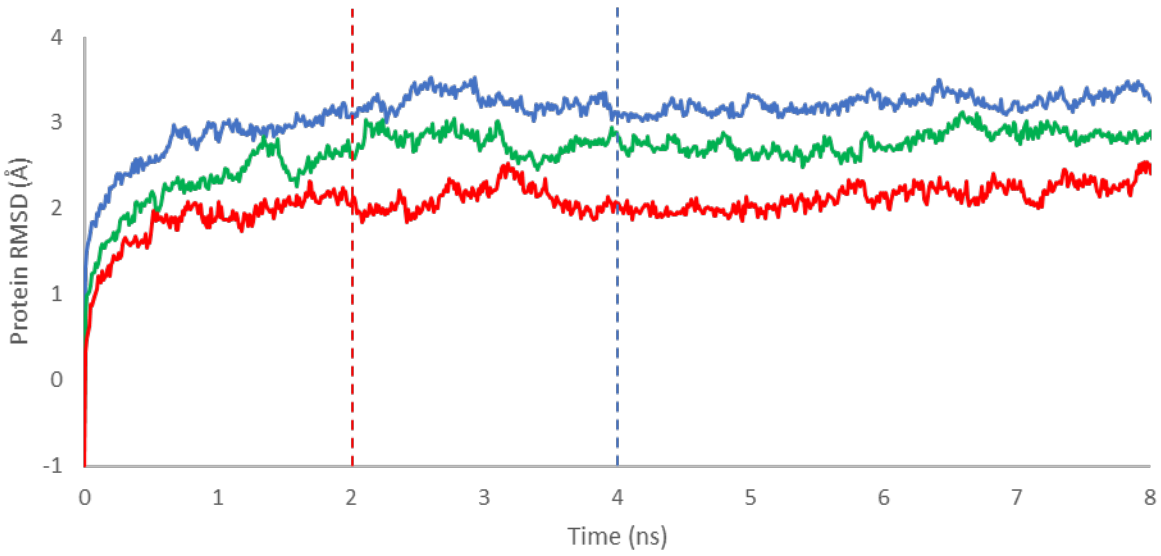

Figure S2 Each MC/MD simulation"converges" (reaches a plateau of intrinsic random motion) after approximately 2 ns (red vertical bar), and beginning at 4 ns (the blue vertical bar) the protein isoform structures do not undergo any significant conformational change during further energy minimization. Data shown are one of three independent 10 ns MC/MD simulations for each PfCRT isoform; HB3 (blue, top), 7G8 (green, middle), and Dd2 (red, bottom). Note the plots have been offset from one another (-0.5 Å for 7G8 and -1.0 Å for Dd2, relative to HB3, c.f. Y axis) for ease of visual comparison.

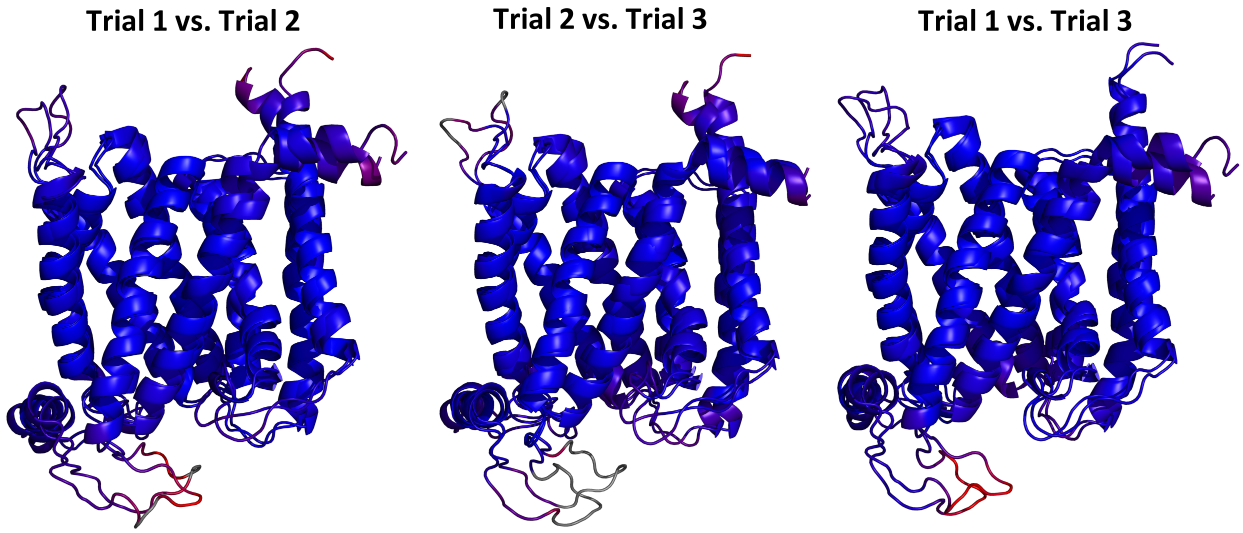

Figure S3. RMSD comparison (summarized in Table 1B) between different individual MC/MD 10 ns trials solving for the HB3^{EMMD} structure (see text).

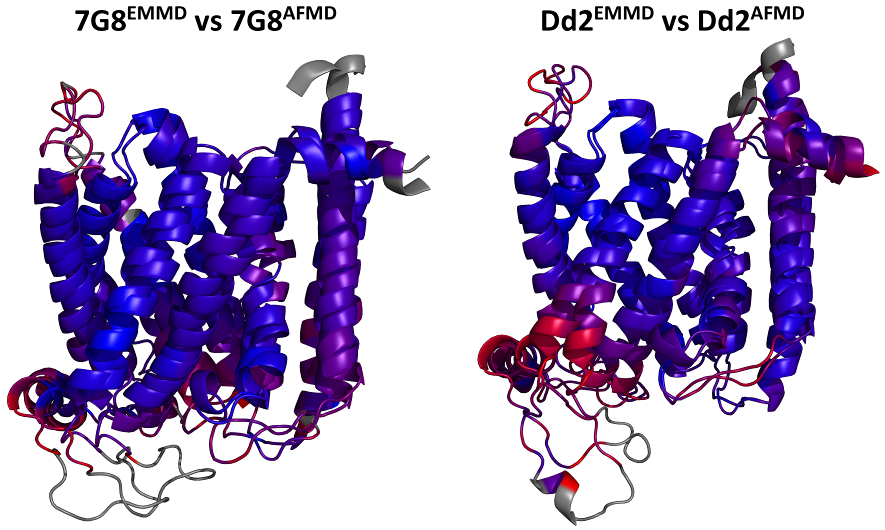

Figure S4. RMSD comparison between **A, left)** 7G8^{EMMD} and 7G8^{AFMD} and **B, right)** Dd2^{EMMD} and Dd2^{AFMD} 10 TM cores (excluding residues 1-46, 405-424). Blue and red indicate the minimum (0.50, 0.34 Å) and maximum (9.45, 6.55 Å) pairwise RMSD in the two comparisons respectively, the average all atom RMSD are 3.07 and 2.11 Å respectively. Similar results are obtained when comparing HB3^{AFMD} to HB3^{EMMD} (c.f. Fig. 2).

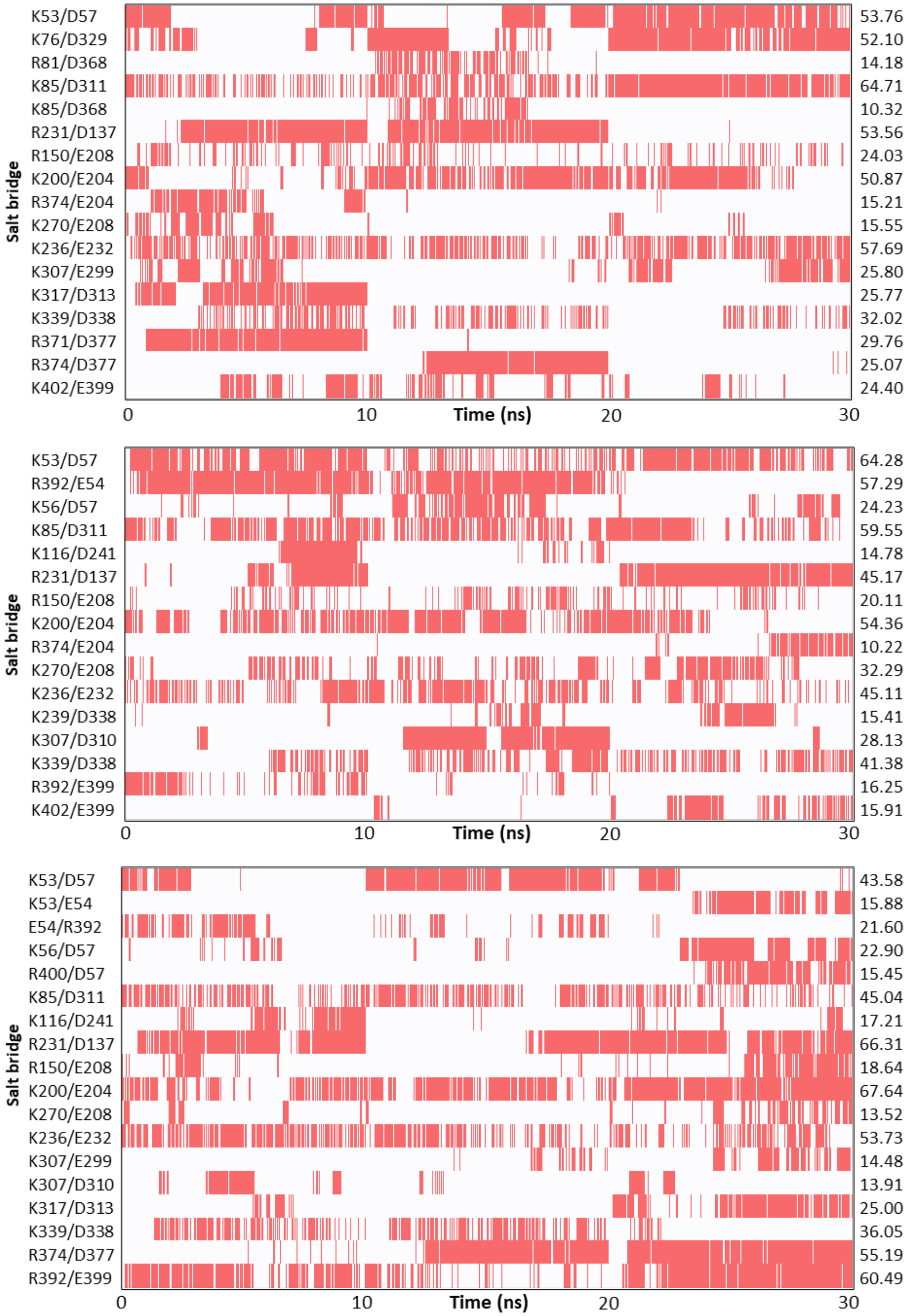

Figure S5. Heatmap of HB3 (top), 7G8 (middle), and Dd2 (bottom) PfCRT SB lifetimes. When the hetero atoms of two interacting residues are ≤ 4 Å apart, the SB exists, as denoted by a vertical

red mark. Data are from 3 separate 10 ns simulations clustered together. On the right side of the heat maps, the lifetime of the interaction is given as a percentage of total frames.

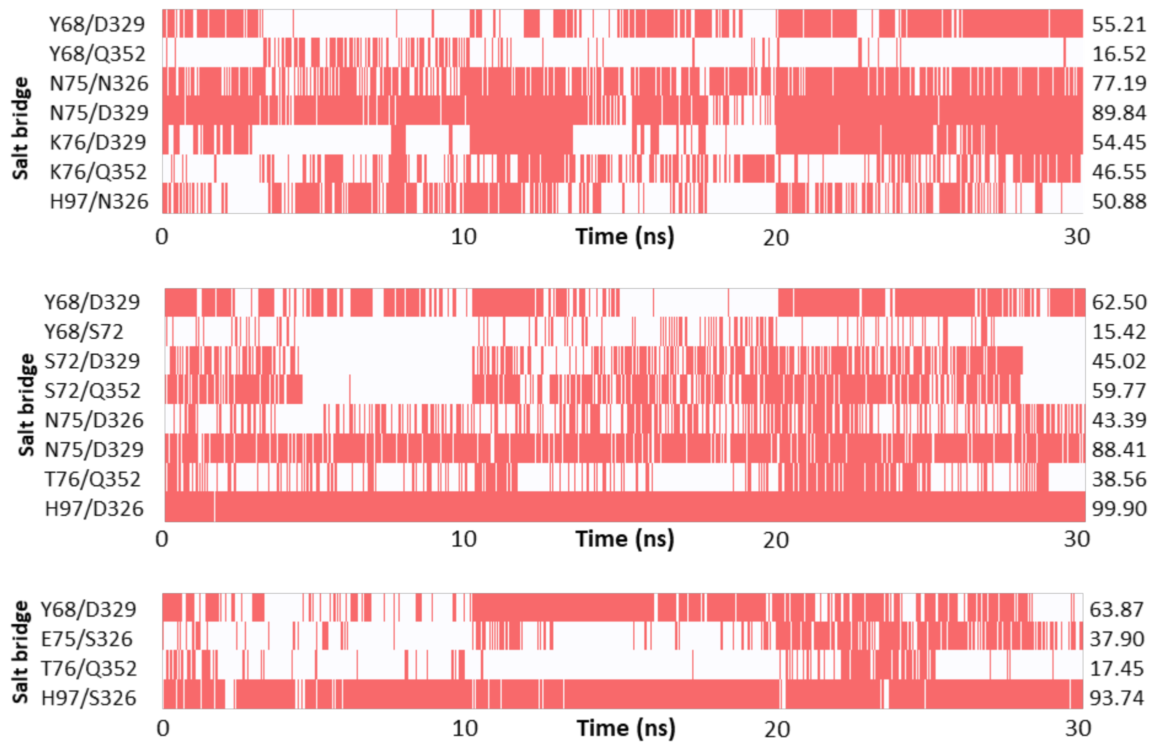

Figure S6. Heatmap of HB3 (top), 7G8 (middle), and Dd2 (bottom) PfCRT SB and HB found in their respective "residue 76 region networks" (c.f. Fig. 5). When two interacting residues are ≤ 4 Å (SB) or ≤ 3.2 Å apart (HB), this is denoted as a vertical red mark. Data are from 3 independent 10 ns simulations clustered together. Interaction lifetime is given as % of total frames (right).

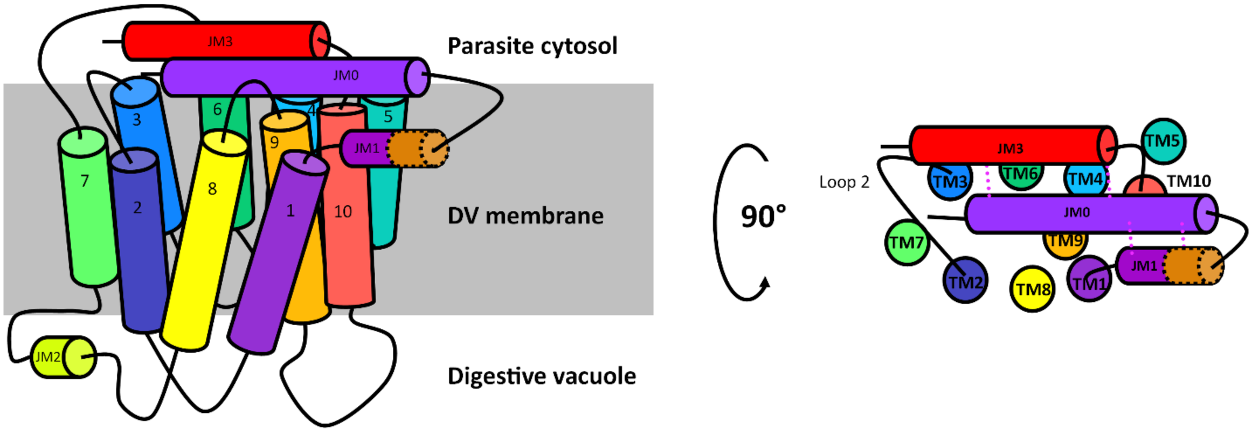

Figure S7. Revised cartoon of PfCRT topology colored as in Kim *et al.*[19] and including a 3 helix "zipper" defined here based on PfCRT^{AFMD} structures. Shown are side (left) or top down views (from the cytosol; right). The 10 TM core helices are numbered 1-10, along with portions of **j**uxta **m**embrane helix 1 (JM1; dark purple & orange) and JM2 (yellow - green), which were previously resolved in Kim *et al* [19]. Additional JM1 structure elucidated here via AF2 (see text) is shown in orange. The cytosolic zipper, consisting of newly defined JM0 (purple), JM1 (dark purple & orange), and JM3 (red) appears to fold directly above the cytosolic opening of the PfCRT central pore after MC/MD energy minimization (see also Fig. 8)

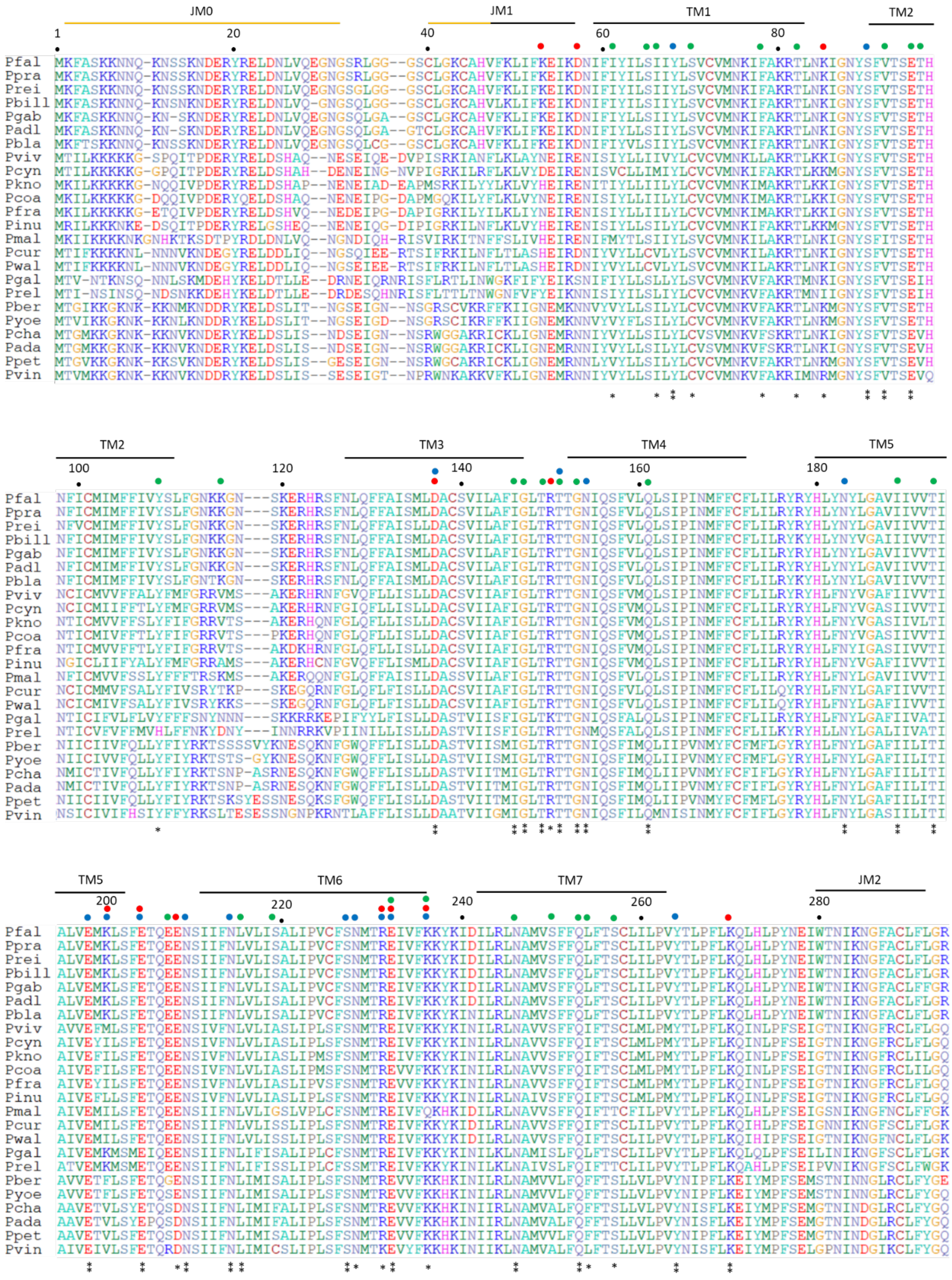
JM0
JM1
TM1
TM2
TM3
TM4
TM5
TM6
TM7
JM2
Pfal
Ppra
Prei
Pbill
Pgab
Padl
Pbla
Pviv
Pcyn
Pkno
Pcoa
Pfra
Pinu
Pmal
Pcur
Pwal
Pgal
Prel
Pber
Pyoe
Pcha
Pada
Ppet
Pvin

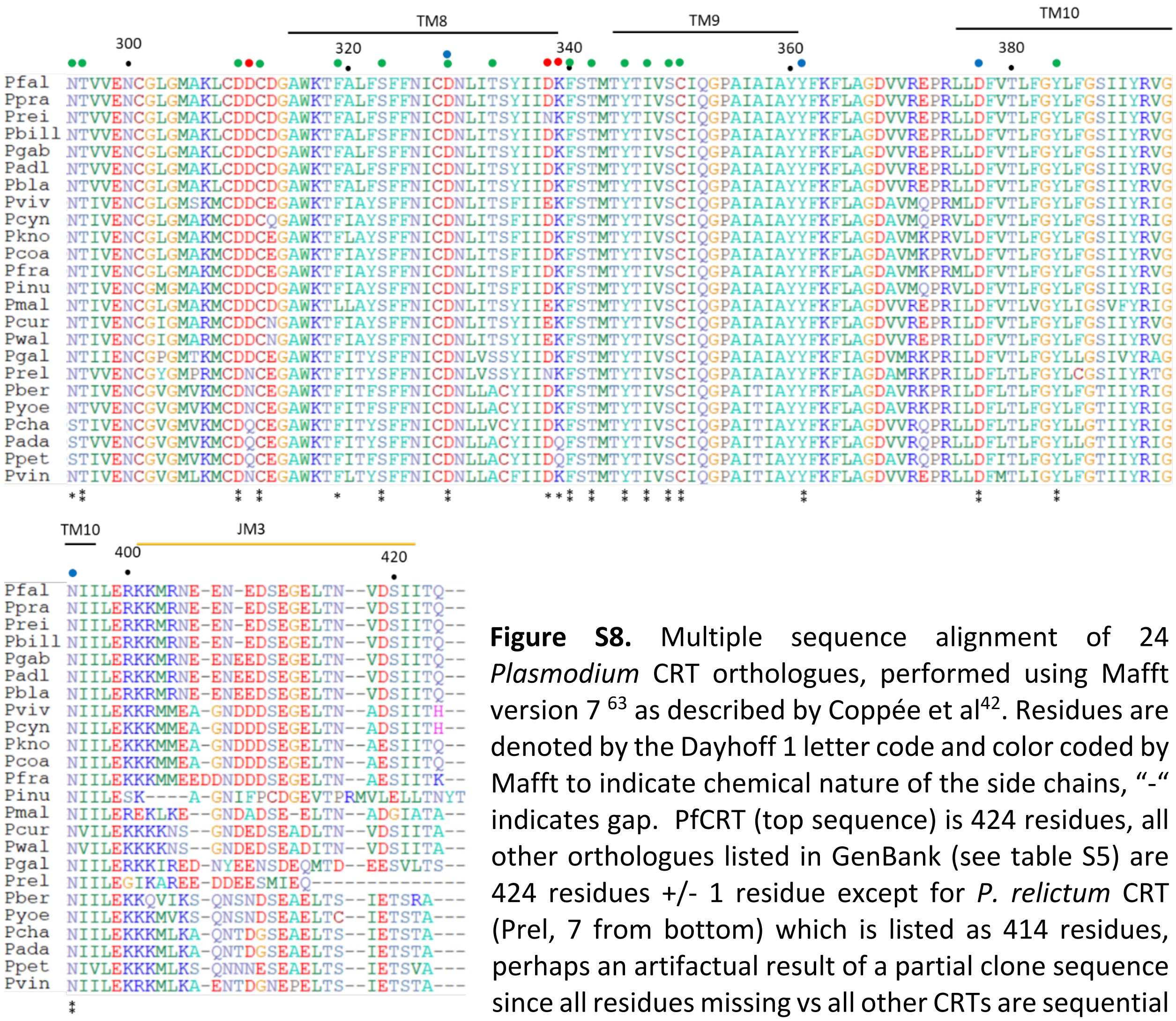

Figure S8. Multiple sequence alignment of 24 *Plasmodium* CRT orthologues, performed using Mafft version 7 [63] as described by Coppée et al[42]. Residues are denoted by the Dayhoff 1 letter code and color coded by Mafft to indicate chemical nature of the side chains, "-" indicates gap. PfCRT (top sequence) is 424 residues, all other orthologues listed in GenBank (see table S5) are 424 residues +/- 1 residue except for *P. relictum* CRT (Prel, 7 from bottom) which is listed as 414 residues, perhaps an artifactual result of a partial clone sequence since all residues missing vs all other CRTs are sequential and C terminal (denoted "-", Prel 413 – 425 relative to Pfal). Black dots indicate PfCRT codon numbers which are found above the black dots, and horizontal lines above the sequences denote previously determined (black; see also Kim *et al.* [19]) or AFMD resolved herein (orange) conformation and topology (see also text and Fig. S7). Colored circles above a given codon indicates the type of interaction in which the residue participates (see also tables S2, S3, S4); red indicates a SB; blue indicates a sidechain – sidechain HB; and green indicates a sidechain – peptide back bone HB, note some residues may participate in more than one type of interaction during protein conformational dynamics (compare tables S2 – S4). When the residue for a given interaction is identical across all 24 orthologues, it is indicated by a double asterisk "⁑" underneath the column of residues at a given position, while residues that are conserved in ≥ 22 orthologues are indicated by a single asterisk "*". Accession numbers can be found in [42].

Supplemental Tables

Table S1. Amino acid differences between the three PfCRT isoforms studied in this paper. Highlighted in green are residues that differ for 7G8 and Dd2 (CQR) vs HB3 (CQS) isoforms.

Cognate strain	Cognate strain	Codon position									Phenotype
Origin		72	74	75	76	220	271	326	356	371	
Honduras	HB3	C	M	N	K	A	Q	N	I	R	CQS
Brazil	7G8	S	M	N	T	S	Q	D	L	R	CQR
Thailand	Dd2	C	I	E	T	S	E	S	T	I	CQR

Table S2. Lifetimes for all SB found at ≤4 Å hetero atom to hetero atom distance for ≥ 10 % of simulation time for at least one isoform (see text). The salt bridges are listed in order of the lowest numbered residue and the residue pairs are in the order of "donor"/"acceptor". Residues that are mutated in different isoforms are *italicized*. Color code denotes lifetime frequency (> 40%, green; 10-40%, yellow; <10%, red).

Salt Bridge	HB3	7G8	Dd2	Location Res #1	Location Res #2
K53/E54	0.00%	6.99%	15.88%	JM1	JM1
K53/D57	53.76%	64.28%	43.58%	JM1	JM1
R392/E54	7.29%	57.29%	21.60%	TM10	JM1
K56/D57	3.50%	24.23%	22.90%	JM1	JM1
R400/D57	0.00%	0.00%	15.45%	**L10**	JM1
K76/D329	52.10%	0.00%	0.00%	TM1	TM8
R81/D368	14.18%	0.00%	0.00%	*TM1*	**L9**
K85/D311	64.71%	59.55%	45.04%	**L1**	**L7**
K85/D368	10.32%	0.80%	5.99%	**L1**	**L9**
K116/D241	5.46%	14.78%	17.21%	**L2**	**L6**
R231/D137	53.56%	45.17%	66.31%	TM6	TM3
R150/E208	24.03%	20.11%	18.64%	**L3**	**L5**
K200/E204	50.87%	54.36%	67.64%	*TM5*	**L5**
R374/E204	15.21%	10.22%	0.00%	**L9**	**L5**
K270/E208	15.55%	32.29%	13.52%	**L7**	**L5**
K236/E232	57.69%	45.11%	53.73%	*TM6*	TM6
K239/D338	3.93%	15.41%	7.39%	**L6**	*TM8*
K307/E299	25.80%	9.12%	14.48%	**L7**	**L7**
K307/D310	0.00%	28.13%	13.91%	**L7**	**L7**
K317/D313	25.77%	0.00%	25.00%	*TM8*	**L7**
K339/D338	32.02%	41.38%	36.05%	*TM8*	*TM8*

R371/D377	29.76%	0.27%	0.00%	**L9**	*TM10*
R374/D377	25.07%	4.59%	55.19%	**L9**	*TM10*
R392/E399	0.13%	16.25%	60.49%	TM10	*TM10*
K402/E399	24.40%	15.91%	1.90%	*TM10*	*TM10*

Table S3. Lifetimes for all HB found at ≤3.2 Å hetero atom to hetero atom distance for ≥ 10 % of simulation time for at least one isoform (see text). The HB are listed in the order of the lowest numbered residue and residue pairs in the order of "HB donor"/"acceptor". Residues that are mutated in different isoforms are *italicized*. Color code denotes lifetime frequency (>50%, green; 10-50%, yellow; <10%, red).

**Hydrogen bond**	**HB3**	**7G8**	**Dd2**
K53/D57	42.94%	64.91%	37.38%
K53/E54	<10%	<10%	13.55%
Y62/E54	36.15%	67.38%	59.15%
R392/E54	35.55%	67.98%	49.97%
N58/E54	29.83%	53.66%	38.22%
K56/D57	<10%	20.77%	18.14%
R400/D57	<10%	<10%	23.87%
N58/E399	<10%	<10%	21.50%
R392/Y62	28.96%	<10%	20.77%
Y68/D329	54.79%	62.08%	63.22%
Y345/Y68	19.71%	21.84%	30.56%
S72/D329	<10%	44.44%	<10%
Q352/*S72*	<10%	55.43%	<10%
N75/*N326*	52.30%	<10%	<10%
N75/D329	87.48%	86.62%	<10%
N326/*N75*	15.01%	<10%	<10%
N75/*D326*	<10%	42.48%	<10%
S326/*E75*	<10%	<10%	37.25%
K76/Q352	35.22%	<10%	<10%
K76/D329	43.08%	<10%	<10%
Q352/*T76*	<10%	20.57%	<10%
K80/Q156	14.31%	15.45%	<10%
K80/Y360	11.78%	<10%	<10%
K80/*T356*	<10%	<10%	11.95%
R81/D368	16.74%	<10%	<10%
T318/T82	<10%	<10%	35.59%
N84/D368	<10%	19.41%	<10%
K85/D311	52.06%	47.74%	46.97%
ILE86/C312	<10%	<10%	17.11%
N88/D310	49.17%	28.16%	39.98%
Y89/T296	11.48%	<10%	23.30%
Y89/D310	52.96%	56.96%	42.74%

N285/Y89	12.78%	<10%	<10%
Y89/C289	<10%	11.52%	10.35%
Y264/S90	57.76%	92.14%	76.96%
S94/S257	14.45%	<10%	<10%
N98/E95	18.14%	21.80%	37.62%
E95/S257	<10%	<10%	10.19%
N326/H97	39.01%	<10%	<10%
H97/*D326*	<10%	91.08%	<10%
H97/*S326*	<10%	<10%	86.02%
N98/S257	17.44%	28.73%	18.74%
K116/D241	<10%	<10%	11.28%
R244/N118	31.76%	<10%	16.74%
H123/Q129	27.43%	11.42%	<10%
S125/N127	22.34%	41.28%	22.20%
S134/Q253	28.10%	<10%	<10%
T256/S134	30.36%	<10%	<10%
S134/T256	11.98%	<10%	12.58%
R231/D137	68.41%	62.85%	72.07%
S227/D137	92.41%	93.18%	91.78%
Q253/D137	<10%	<10%	10.32%
R150/E208	71.67%	50.97%	37.45%
N214/T151	80.59%	81.72%	82.92%
T152/E207	81.32%	52.13%	47.40%
T205/T152	15.78%	13.95%	<10%
T152/S202	<10%	18.24%	12.12%
N209/T152	<10%	12.78%	11.19%
N154/E198	94.97%	94.71%	94.81%
S157/E198	48.60%	31.76%	54.06%
N167/T346	36.62%	<10%	<10%
N167/T342	31.39%	<10%	<10%
T342/N167	10.39%	10.39%	35.72%
C225/N167	14.58%	24.07%	20.91%
Y391/M168	<10%	13.98%	<10%
Y177/E232	28.93%	33.82%	42.34%
Y179/M343	11.12%	35.92%	19.97%
H180/N183	11.19%	<10%	<10%
N183/N395	50.73%	57.99%	81.49%
N183/Y391	<10%	15.01%	23.50%
K200/E204	53.30%	51.26%	63.65%
R374/E204	18.91%	11.95%	<10%
K270/E208	11.25%	27.06%	14.21%
N214/N209	85.39%	75.43%	83.32%
N209/N214	53.10%	34.92%	59.79%
R231/S227	38.08%	20.61%	25.40%
R231/N228	53.93%	53.99%	66.98%

R231/S334	22.20%	16.25%	15.01%
K236/E232	73.24%	54.46%	62.28%
T342/E232	27.33%	62.52%	28.63%
S341/E232	<10%	36.58%	21.11%
N246/S334	41.21%	26.33%	18.51%
S334/N246	38.98%	32.59%	68.28%
N246/N330	<10%	16.81%	19.94%
S257/Q253	11.25%	17.31%	<10%
T256/Q253	21.54%	31.56%	18.61%
N285/T296	14.35%	<10%	<10%
N295/D313	29.03%	51.66%	28.23%
T296/D310	47.10%	85.09%	79.76%
K307/E299	21.07%	<10%	10.99%
K307/D310	<10%	18.61%	<10%
D310/C312	20.81%	24.80%	19.61%
W316/D313	<10%	<10%	12.45%
K317/D313	<10%	<10%	13.12%
Y345/T333	35.59%	34.52%	14.31%
K339/D338	25.37%	31.99%	30.33%
T344/S341	63.25%	51.50%	29.36%
Y361/D377	98.14%	96.84%	98.27%
R371/D377	49.77%	46.21%	<10%
R374/D377	26.03%	15.15%	56.96%
S388/Y384	11.09%	<10%	<10%
R392/E399	<10%	36.58%	77.50%
K402/E399	17.24%	11.05%	<10%

Table S4. Lifetimes for all side chain-backbone peptide bond HB found at ≤ 3.2 Å hetero atom to hetero atom distance for ≥ 10 % of simulation time for at least one isoform (see text). The HB are listed in the order of the lowest numbered residue and residue pairs are in the order of "HB donor"/"acceptor". Residues that are mutated in different isoforms are *italicized*. The residue immediately preceding the relevant peptide backbone is indicated in **bold.** Color code denotes lifetime frequency (> 50%, green; 10-50%, yellow; <10%, red).

Hydrogen Bond	HB3	7G8	Dd2
N58/**E54**	28.26%	24.30%	<10%
S65/**I61**	70.31%	67.58%	74.13%
S70/**I66**	75.60%	69.94%	83.75%
S70/**I67**	10.25%	16.15%	<10%
C72/**Y68**	18.44%	<10%	<10%
S72/**Y68**	<10%	18.38%	<10%
T76/***S72***	<10%	73.74%	<10%
T76/***C72***	<10%	<10%	14.68%
Q352/***C72***	<10%	<10%	35.59%
T76/**V73**	<10%	<10%	12.62%
T82/**F78**	94.17%	73.64%	76.83%
N84/**K80**	20.37%	16.08%	21.67%
R81/**L365**	29.89%	28.53%	49.63%
R81/**F364**	12.08%	15.71%	<10%
S90/**G87**	16.71%	<10%	17.04%
N88/**L308**	43.68%	16.31%	26.80%
N88/***Q271***	<10%	29.03%	<10%
**Y89**/N88	<10%	19.31%	<10%
**L308**/N88	<10%	21.77%	18.38%
N88/***E271***	<10%	<10%	13.42%
T93/**Y89**	31.42%	18.71%	<10%
S94/**S90**	21.94%	41.28%	25.33%
T93/**S90**	20.71%	<10%	14.55%
**F91**/Y264	30.06%	37.45%	39.55%
T96/**V92**	94.97%	97.17%	97.74%
N98/**S94**	33.72%	<10%	<10%
E95/**L254**	89.65%	97.87%	85.59%
C101/**H97**	44.01%	35.82%	42.08%
S110/**F106**	47.30%	47.50%	36.52%
Y109/**K116**	54.73%	53.50%	75.30%
N118/**H123**	<10%	<10%	11.85%
R124/**E121**	<10%	<10%	14.68%
R244/**R124**	23.10%	<10%	34.65%
**F126**/S125	<10%	14.35%	<10%
S134/**F130**	28.10%	62.12%	34.69%
S134/**F131**	12.35%	15.38%	37.02%

Q253/**S134**	35.52%	35.79%	26.53%
T256/**S134**	21.54%	31.72%	36.88%
C139/**M135**	32.09%	49.03%	36.02%
S140/**D137**	10.92%	12.92%	20.04%
S140/***A220***	62.58%	<10%	<10%
S140/***S220***	<10%	34.59%	27.50%
T149/**I146**	97.20%	96.94%	61.32%
T151/**G147**	81.79%	83.36%	84.79%
R150/**F268**	<10%	19.51%	21.87%
Q156/**T151**	72.24%	70.97%	46.11%
**T152**/N209	33.62%	40.61%	36.85%
**G153**/E207	76.03%	52.83%	51.40%
S157/**G153**	<10%	14.01%	<10%
Q161/**S157**	29.46%	27.93%	30.63%
S163/**L160**	43.87%	12.12%	40.38%
C350/**L160**	10.22%	<10%	<10%
Q161/**C350**	73.87%	65.18%	63.25%
N167/**S163**	41.84%	83.99%	89.21%
C171/**N167**	44.94%	46.24%	54.29%
R176/**L173**	<10%	12.48%	<10%
**H180**/N183	62.52%	62.32%	33.39%
**L181**/H180	10.82%	12.58%	<10%
T193/**I189**	96.77%	96.94%	97.37%
S202/**E198**	12.45%	24.20%	26.63%
S202/**M199**	23.57%	19.94%	11.92%
T205/**L201**	15.91%	<10%	<10%
T205/**S202**	12.88%	28.43%	<10%
**E207**/T205	<10%	<10%	12.08%
S219/**L215**	87.02%	82.19%	81.06%
S220/**L217**	<10%	98.40%	98.83%
C225/**L221**	16.51%	<10%	<10%
T230/**F226**	51.90%	68.84%	41.51%
R231/**S227**	18.04%	<10%	11.12%
**T342**/E232	90.05%	81.82%	84.19%
K236/**F340**	71.57%	66.61%	73.24%
K239/**K236**	23.10%	17.61%	22.87%
K236/**D338**	<10%	12.82%	<10%
**R244**/D241	23.07%	<10%	<10%
**L243**/D241	31.69%	21.77%	13.45%
N246/**I242**	45.31%	19.41%	<10%
N246/**N330**	13.02%	<10%	20.57%
S250/**N246**	51.23%	58.66%	53.66%
T256/**F252**	<10%	<10%	11.62%
S257/**Q253**	73.87%	53.00%	52.63%
C258/**F255**	11.65%	14.75%	12.68%

T265/**P262**	44.24%	73.24%	80.93%
H273/**Y264**	31.56%	88.32%	22.74%
**L308**/*Q271*	<10%	19.07%	<10%
N282/**P275**	<10%	<10%	12.88%
T281/**N277**	17.98%	<10%	<10%
N282/**E278**	33.29%	<10%	12.32%
T281/**E278**	17.94%	31.03%	30.39%
N282/**I279**	26.73%	65.41%	34.95%
N295/**D310**	13.18%	<10%	<10%
**C312**/N295	73.00%	72.87%	74.47%
**D313**/N295	<10%	<10%	15.88%
**T296**/D310	68.48%	88.72%	74.83%
**V297**/D310	27.73%	17.74%	22.10%
**C309**/C301	<10%	12.55%	<10%
**G302**/M305	18.38%	31.92%	24.50%
K307/**L308**	<10%	15.28%	<10%
**G314**/D311	10.09%	<10%	15.68%
W316/**D313**	15.11%	<10%	12.15%
T318/**G314**	49.70%	<10%	32.29%
S323/**F319**	85.45%	73.34%	56.16%
S323/**A320**	<10%	<10%	32.42%
C328/**F324**	30.96%	31.62%	20.21%
N330/***N326***	43.31%	<10%	<10%
N330/***D326***	<10%	35.25%	<10%
N330/***S326***	<10%	<10%	32.52%
T333/**D329**	88.55%	90.31%	91.91%
S334/**N330**	38.68%	43.71%	15.61%
**T344**/S341	44.71%	27.40%	36.98%
**M343**/S341	<10%	13.75%	<10%
T346/**T342**	91.94%	14.38%	41.84%
Y391/**M343**	<10%	<10%	18.81%
S349/**Y345**	57.49%	58.49%	71.50%
C350/**T346**	38.45%	41.21%	40.18%
Y384/**I347**	98.27%	98.24%	97.60%
Q352/**S349**	41.48%	13.65%	<10%
T356/**Q352**	<10%	<10%	96.01%
R371/**A357**	17.14%	<10%	<10%
**R374**/Y361	15.08%	<10%	26.73%
K363/**Y361**	<10%	19.14%	<10%
**R374**/D377	18.18%	23.44%	16.64%
T380/**L376**	24.83%	33.56%	34.95%
T380/**D377**	27.53%	27.63%	29.26%
S388/**Y384**	41.21%	19.31%	25.77%

Table S5 Key for 4 letter abbreviations of the 24 *Plasmodium spp.* shown in figure S8. Table based on Coppée et al [42] wherein the accession number for each orthologue and alignment methodology can also be found.

**Species**	**Abbreviation**
Plasmodium falciparum	Pfal
Plasmodium praefalciparum	Ppra
Plasmodium reichenowi	Prei
Plasmodium billcollinsi	Pbill
Plasmodium gaboni	Pgab
Plasmodium adleri	Padl
Plasmodium blacklocki	Pbla
Plasmodium vivax	Pviv
Plasmodium cynomolgi	Pcyn
Plasmodium knowlesi	Pkno
Plasmodium coatneyi	Pcoa
Plasmodium fragile	Pfra
Plasmodium inui	Pinu
Plasmodium malariae	Pmal
Plasmodium ovale curtisi	Pcur
Plasmodium ovale wallikeri	Pwal
Plasmodium gallinaceum	Pgal
Plasmodium relictum	Prel
Plasmodium berghei	Pber
Plasmodium yoelii	Pyoe
Plasmodium chabaudi	Pcha
Plasmodium chabaudi adami	Pada
Plasmodium vinckei petteri	Ppet
Plasmodium vinckei	Pvin

STRUCTURE PDB FILES available from the authors (correspondence to PDR)

FI NAME	SIZE
**MC/MD energy minimized structures based on the cryo-EM 7G8 structure**	
HB3_EMMD.pdb	471 KB
7G8_EMMD.pdb	471 KB
Dd2_EMMD.pdb	470 KB
**AlphaFold output structures:**	
HB3_AF.pdb	548 KB
7G8_AF.pdb	547 KB
Dd2_AF.pdb	546 KB
**AlphaFold MC/MD energy minimized structures**	
HB3_AFMD.pdb	553 KB
7G8_AFMD.pdb	552 KB
Dd2_AFMD.pdb	551 KB
**EMMD structures with CQ docked in site A**	
HB3_EMMD_CQA.pdb	494 KB
7G8_EMMD_CQA.pdb	493 KB
Dd2_EMMD_CQA.pdb	493 KB
**EMMD structures with CQ docked in site B**	
HB3_EMMD_CQB.pdb	480 KB
7G8_EMMD_CQB.pdb	493 KB
Dd2_EMMD_CQB.pdb	493 KB
